# Crosstalk between regulated necrosis and micronutrition, bridged by reactive oxygen species

**DOI:** 10.3389/fnut.2022.1003340

**Published:** 2022-09-23

**Authors:** Lei Zhang, Jinting Liu, Ziyan Dai, Jia Wang, Mengyang Wu, Ruicong Su, Di Zhang

**Affiliations:** ^1^College of Veterinary Medicine, Jilin Agricultural University, Changchun, China; ^2^College of Animal Science and Technology, Jilin Agricultural University, Changchun, China; ^3^Jilin Provincial Engineering Research Center of Animal Probiotics, Jilin Agricultural University, Changchun, China

**Keywords:** necroptosis, ferroptosis, pyroptosis, selenium, iron, zinc, vitamins, reactive oxygen species

## Abstract

The discovery of regulated necrosis revitalizes the understanding of necrosis from a passive and accidental cell death to a highly coordinated and genetically regulated cell death routine. Since the emergence of RIPK1 (receptor-interacting protein kinase 1)-RIPK3-MLKL (mixed lineage kinase domain-like) axis-mediated necroptosis, various other forms of regulated necrosis, including ferroptosis and pyroptosis, have been described, which enrich the understanding of pathophysiological nature of diseases and provide novel therapeutics. Micronutrients, vitamins, and minerals, position centrally in metabolism, which are required to maintain cellular homeostasis and functions. A steady supply of micronutrients benefits health, whereas either deficiency or excessive amounts of micronutrients are considered harmful and clinically associated with certain diseases, such as cardiovascular disease and neurodegenerative disease. Recent advance reveals that micronutrients are actively involved in the signaling pathways of regulated necrosis. For example, iron-mediated oxidative stress leads to lipid peroxidation, which triggers ferroptotic cell death in cancer cells. In this review, we illustrate the crosstalk between micronutrients and regulated necrosis, and unravel the important roles of micronutrients in the process of regulated necrosis. Meanwhile, we analyze the perspective mechanism of each micronutrient in regulated necrosis, with a particular focus on reactive oxygen species (ROS).

## Introduction

Regulation of tissue homeostasis by programmed cell death (PCD) is a fundamental process with broadly physiological and pathological implications. As one of the most investigated PCDs, apoptosis is characterized as a strictly regulated and caspase-dependent cell death routine, which is essential for the maintenance and repair of tissues (1). Dysfunction of apoptosis contributes to a broad range of developmental abnormalities and diseases (2, 3). Unlike necrosis, apoptosis keeps its membrane integrity, which retains its intracellular content and does not trigger inflammation. On the other hand, necrosis has been described as an unregulated and accidental cell death, triggered by extracellular signals, such as cytokines, microbial infections, or ischemia/reperfusion (I/R) (4). The rapid rupture of plasma membrane prompts the release of inflammatory cellular contents into the microenvironment, which activates immune system and initiates inflammatory response (5–7). Unrestrained activation of necrosis contributes to various diseases and injuries in multiple tissues, like in kidney (8) and liver (9).

Recent advances in cell biology revolutionize the nomenclature of necrosis by identifying that at least some forms of necrosis are reversibly PCD processes, referred as regulated necrosis. Three forms of regulated necrosis, necroptosis, ferroptosis, and pyroptosis, are discovered and studied in detail. Among them, tumor necrosis factor α (TNFα)-triggered necroptosis is the first established one, which can be inhibited by a small molecule, necrostatin-1 (Nec-1) *via* targeting receptor-interacting protein kinase 1 (RIPK1) (10). Ferroptosis is another unique form of regulated necrosis that is executed by iron-derived lipid peroxidation. Labile iron or iron-containing lipoxygenases (LOXs)-produced lipid peroxides facilitate the formation of structured lipid pores on the plasma membrane, leading to cell death (11, 12). Pyroptosis, triggered by activation of inflammasome, is a more recently identified form of regulated necrosis, stimulated by microbial infections and non-infectious factors (13, 14).

Reactive oxygen species (ROS) are O_2_-derived free radical (such as superoxide anion and hydroxyl radical) and non-radical species (such as hydrogen peroxide) (15). As byproducts, ROS are produced by partial reduction of oxygen, proceeding from either endogenous mitochondrial oxidative phosphorylation, or exogenous sources, such as heavy metals and radiation (16). Normally, excess ROS are rapidly removed by cellular antioxidant defense system. However, once the redox balance is impaired, the overwhelming ROS-caused oxidative stress can induce damage to cellular macromolecules directly or indirectly, which leads to a variety of pathophysiological conditions, including regulated necrosis. For instance, mitochondria-derived ROS promoted autophosphorylation of RIPK1, which enabled RIPK1 to recruit RIPK3 to form necrosome (RIPK1/RIPK3), initiating TNFα-mediated necroptosis in colon cancer cells (17). Iron-dependent lipid peroxidation is a major driver to ferroptosis (18). Wang et al. also found that ROS promoted NOD-like receptor pyrin domain-containing protein 3 (NLRP3) inflammasome activation and caspase-1-dependent gasdermin D (GSDMD) cleavage, leading to pyroptotic cell death in macrophages (19).

Micronutrients are elements that consist of minerals and vitamins. Although they appear in trace amount, micronutrients are indispensable to life functions, such as metabolism, cell growth, development, and differentiation (20, 21). Recent studies have disclosed the critical role of micronutrients in regulation of regulated necrosis process (22, 23). Interestingly, ROS are found important in the crosstalk between micronutrients and regulated necrosis. In this review, we illustrate the critical roles of major micronutrients in the biological process of regulated necrosis, with a focus on ROS as a link that bridges the interplay between micronutrients and regulated necrosis.

## Molecular mechanisms of regulated necrosis

### Necroptosis

**FIGURE 1:**
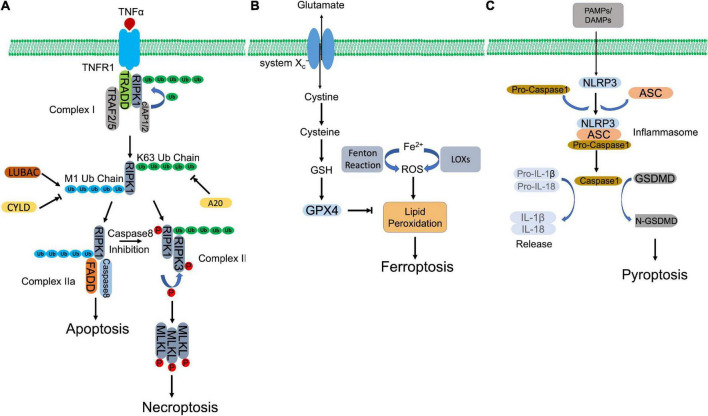
Molecular mechanisms of regulated necrosis. **(A)** Necroptosis. Activation of TNFR1 by TNFα induces the formation of complex I, which contains RIPK1, TRADD, TRAF2/5, and cIAP1/2. Then, RIPK1 is ubiquitinated by cIAP1/2 to form K63-ubiquitin chains, which is removed by A20. The formed K63 uboquitination recruits LUBAC to complex I, where LUBAC catalyzes the formation of M1-ubiquitin chain on RIPK1, which is removed by CYLD. K63 ubiquitination of RIPK1 promotes the formation of necrosome, which subsequently phosphorylates MLKL to initiate necroptosis. Whereas, M1 ubiquitination induces the assembly of complex IIa, which initiates apoptosis. When caspase 8 is inhibited, necroptosis is triggered *via* activation of necrosome. **(B)** Ferroptosis. Aberrant buildup of ROS induces lipid peroxidation, which leads to ferroptosis. Intracellular ferrous ion (Fe^2+^) catalyzes lipid peroxidation *via* Fenton reaction and LOXs. GPX4, in turn, hydrolyses lipid peroxides, converting them to corresponding lipid alcohols. The antioxidant activity of GPX4 requires the participation of GSH, whose synthesis depends on cystine/glutamate antiporter system X_c_^–^. inhibition of GPX4 or system X_c_^–^ initiates ferroptosis. **(C)** Pyroptosis. PAMPs and DAMPs activate inflammasome assembly. Inflammaspme sensor (NLRP3) interacts with ASC, which recruits pro-caspase 1. Pro-caspase 1 is activated by autocleavage. Activated casapse 1 not only cleaves GSDMD to induce pyroptosis, but also processes the precursors of IL-1β/IL-18 to mature IL-1β/IL-18, which are released through the pores formed by N-GSDMD.

Necroptosis is characterized with morphological features, including organelle swelling, plasma membrane rupture, and leakage of intracellular components (24). In consistent with uncontrolled necrosis, necroptosis also triggers inflammatory response (25). However, unlike necrosis, necroptosis can be blocked by a specific inhibitor of RIPK1, Nec-1 (10). The activation of necroptosis pathway has been implicated in many human pathologies, such as I/R injuries, inflammatory bowel disease (IBD) and neurodegenerative disease (24). Necroptosis can be stimulated by a plethora of stimuli, including members of the TNF receptor (TNFR) superfamily (10, 26), pattern recognition receptors (PRRs) (27), T cell receptors (TCRs) (28), multiple chemotherapeutic drugs (29, 30), and environmental stress such as hypoxia (31). Here, we illustrate necroptotic signaling pathway through most investigated TNFα-TNFR1 axis (Figure 1A).

TNFR1 ligation by TNFα recruits key regulator of necroptosis, RIPK1, together with other associated proteins, including TNFR-associated death domain (TRADD), cellular inhibitor of apoptosis protein 1/2 (cIAP1/2), and TNFR-associated factor 2/5 (TRAF2/5), forming a membrane-bound multimeric protein complex, complex I (32–34). Within the complex, RIPK1 is polyubiquitinated by E3 ligases, cIAP1/2, forming a linear Lys-63 (K63) ubiquitin modification that inhibits RIPK1 and promotes NF-κB dependent cell survival (35, 36). Meanwhile, K63 ubiquitin chain also recruits linear ubiquitination (M1-Ubi) assembly complex (LUBAC) and CYLD (a complex of deubiquitinating enzyme), which are responsible to assemble and disassemble the Met 1 (M1)-ubiquitin linear chain on RIPK1, respectively (37–39). The different types of ubiquitin modification or M1/K63 ubiquitination ratio regulates the activation of RIPK1. A higher K63 ubiquitination coordinated by cIAP1/2 and CYLD activates RIPK1 and directs cell to TNFα-mediated necroptotic cell death, while a higher M1 ubiquitination coordinated by LUBAC and A20 (a deubiquitinase to remove K63-linear chain, recruited by M1-ubiquitin chain) protects cells against apoptosis and necroptosis after TNFα stimulation (40–42). Activated RIPK1 recruits FAS-associated death domain protein (FADD) and caspase-8 to form complex IIa. Within the complex, caspase-8 cleaves RIPK1, leading to pro-apoptotic caspase activation and apoptosis (39). Caspase-8 inhibition or deficiency rescues RIPK1, which promotes necroptosis by formation of complex IIb, consisting of RIPK1 and RIPK3 (43, 44). Autophosphorylated RIPK1 recruits and activates RIPK3 *via* phosphorylation (10, 45). The heterodimer of RIPK1 and RIPK3, or necrosome, subsequently phosphorylates the executioner, mixed-lineage kinase domain-like pseudokinase (MLKL) (46). Phosphorylated MLKL then oligomerizes and transmits to the plasma membrane, impairing membrane integrity by forming structured pores and leading to cell death (47).

### Ferroptosis

Ferroptosis is an iron-dependent and lipid peroxidation-reliant regulated necrosis that was firstly described by Dr. Stockwell’s group in 2012 (11). It depicts a necrosis-like morphological changes and represents two biochemical characteristics, iron accumulation, and lipid peroxidation, two processes of which are interdependent (Figure 1B). Free iron (Fenton reaction) or iron-containing LOXs catalyzes lipid peroxidation, which is essential for the execution of ferroptosis by forming structured pores on the plasma membrane. Polyunsaturated fatty acids (PUFAs) are vulnerable to be attacked by iron-originated free radicals. The propagated lipid peroxides generate a myriad of secondary products, such as breakdown products of lipid peroxides and oxidized proteins, ultimately rupturing organelles and cell membrane (48).

Glutathione peroxidase 4 (GPX4), as a lipid hydroperoxidase, maintains the endogenous redox balance by reducing phospholipid hydroperoxides to their corresponding phospholipid alcohol (48). Glutathione (GSH) as a potent reductant, provides the required two electrons to promote GPX4-mediated lipid hydroperoxide reduction (48, 49). To synthesize GSH, cells uptake cysteine by cystine/glutamate antiporter system x_c_^–^, following two ATP-requiring enzymatic steps, formation of γ-glutamylcysteine by glutamate and cysteine, and formation of GSH by γ-glutamylcysteine and glycine (50, 51). RAS-selective lethal 3 (RSL3) directly binds the active site of GPX4 and inhibits its antioxidant activity, while erastin blocks cystine/glutamate antiporter system x_c_^–^ that inhibits the antioxidant activity of GPX4 indirectly (11, 52). Both compounds accumulate iron-dependent lipid peroxides and initiate ferroptosis.

### Pyroptosis

Pyroptosis defends against extracellular pathogen invasion by eliminating the infected cell and triggering inflammatory response. Although it represents some similarities as apoptosis, such as DNA damage and chromatin condensation, pyroptosis is distinct from other forms of cell death morphologically and mechanistically. Unlike apoptosis, pyroptosis is characterized by caspase-1-dependent, gasdermin-mediated rapid plasma membrane rupture and associated proinflammatory intracellular content release (Figure 1C). Inflammasome assembly initiates activation of canonical pyroptotic pathway. Inflammasome is a multiprotein complex that consists of three components, sensors, adaptor, and pro-caspase-1. Sensors are PRRs, which recognize microorganism or host-derived danger signals, designated as pathogen-associated molecular patterns (PAMPs) and danger-associated molecular patterns (DAMPs), and trigger formation of inflammasome. PAMPs and DAMPs activate inflammasome sensors, including Nod-like receptor (NLR) family pyrin domain containing 3 (NLRP3), NLR family caspase activation and recruitment domain (CARD) containing 4 (NLRC4), NLR family pyrin domain-containing 1B (NLRP1B), absent in melanoma 2 (AIM2), and Pyrin (53, 54). Some receptors, like NLRP3, interacts with pro-caspase-1, with the coordination of ASC, an adopter protein that reserves a caspase activation and recruitment domain (CARD) (55). Other receptors, such as NLRC4, recruit pro-caspase-1 with their own CARD domain (55). Within the inflammasome, pro-caspase-1 is auto-cleaved and activated, which in turn hydrolyzes GSDMD, producing N-terminus and C-terminus of GSDMD. N-GSDMD oligomerizes, perforates plasma membrane and induces pyroptotic cell death (56–58). In addition, mature inflammatory cytokines, interleukin-1β (IL-1β) and interleukin (IL-18), cleaved by caspase-1, are released *via* N-DSDMD structured pores (58).

In addition to caspase-1-GSDMD axis, multiple stimuli also trigger pyroptosis through activating other caspases and their targeted GSDMs. Gram-negative bacteria-derived lipopolysaccharide (LPS) activated caspase-4/5/11, which subsequently cleaved GSDMD, leading to pyroptosis (59, 60). Yersinia infection induced pyroptosis through caspase-8-mediated GSDMD cleavage (61, 62). Chemotherapeutics were also observed that promoted caspase-3-dependent GSDME cleavage and induced pyroptosis by forming N-terminus of GSDME structured pores in tumor cells (63).

## Micronutrients and regulated necrosis

### Selenium and regulated necrosis

Selenium (Se) is an essential trace element, which is fundamentally involved in various biological functions. Daily diary provides Se from food, such as cereals, grains, and vegetables (64). A 50 μg/day intake of Se is estimated adequate to support normal cellular functions, whereas a significant higher intake of Se (350–700 μg/day) is toxic (64). Se deficiency also attributes to multiple pathophysiological conditions, such as heart disease, neuromuscular disorder, cancer, male infertility, and inflammation (65, 66). Identification of selenocysteine (Sec) and associated selenoproteins further reveals the importance of Se in physiological process.

#### Biosynthesis of selenoprotein

Se-containing protein, designated as selenoprotein, is biosynthesized through translationally incorporating Sec *via* a specific codon UGA, which is normally a termination codon during regular translational process (67). The *cis*-acting Sec insertion sequence (SECIS) element, a stem-loop-like structure in the 3′ untranslated region of selenoproteins, facilitates the incorporation of this rare amino acid in vertebrates (68). SECIS can be recognized and bond by SECIS binding protein 2 (SPB2) (69). Together with other co-factors assembled on SECIS of selenoprotein mRNA, they switch the UGA from a stop codon to Sec insertion signal (70, 71). The biosynthesis of Sec is executed on its own tRNA, tRNA^Sec^. During the process, seryl-tRNA synthetase catalyzes the conjugation of serine with tRNA^Sec^, which is further phosphorylated by phosphoseryl-tRNA kinase, yielding phospho-serine (P-Ser-tRNA^Sec^) (72). Meanwhile, selenophosphate synthetase 2 (SEPHS2) converts selenite to selenophosphate, which is incorporated in P-Ser-tRNA^Sec^ *via* Sep(O-phosphoserine) tRNA:Sec [selenocysteine] tRNA synthase (SEPSECS), then hydrolyzed, forming Sec-tRNA^Sec^ (23).

As a key feature, most selenoproteins contain Sec residues as the enzymatic site to catalyze redox reaction (73). Currently, 25 selenoproteins are found in human selenoproteome, and majority of them are involved in maintaining cellular redox homeostasis (65, 74). Glutathione peroxidases (GPXs) are important components among the selenoproteins. Eight GPX paralogs have been identified in mammals. Five of them (GPX1, GPX2, GPX3, GPX4, and GPX6) reserve a Sec residue as active site, which is replaced by Cys in the other three GPX homologs (GPX5, GPX7, and GPX8) (75). As the most abundant selenoproteins, GPX1 and GPX4 express ubiquitously, while GPX1 exists in cytoplasm, and GPX4 is found in cytoplasm, mitochondria, and nucleus (75, 76). GPX3 is secreted to plasma by kidney in a glycosylated form, whose activity can be used to evaluate the Se status in the organism (75, 77). GPX2 presents in the epithelial cells of gastrointestinal tract or epithelial tissue, such as lung and liver (75, 78). GPX6 is only found in the olfactory epithelia and embryonic tissues (75, 79). Another major part of selenoproteins is thioredoxin reductases (TXNRDs), which contain a Sec residue at the penultimate position of the C-terminal (80, 81). Three members of TXNRDs have been discovered. TXNRD1 and TXNRD2 are ubiquitously found in the cytoplasm and mitochondria, while TXNRD3 is restrictedly expressed in specific tissues, such as gastrointestinal tract (75, 82).

#### Selenium and necroptosis

**FIGURE 2:**
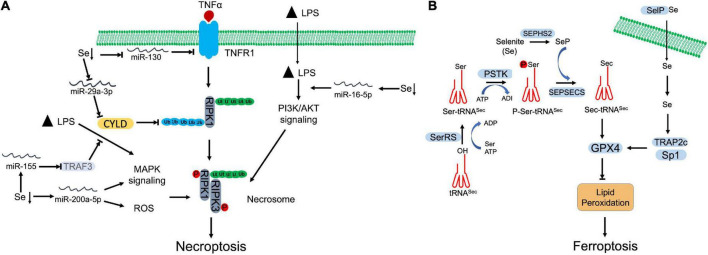
Selenium (Se) antagnized regulated necrosis. **(A)** Necroptosis. Se deficiency promotes necroptosis by manipulating microRNAs, which target specific proteins involving in necroptosis signaling, MAPK signaling, and PI3K/AKT signaling. **(B)** Ferroptosis. To begin the synthesis of selenocysteine (Sec), seryl-tRNA synthetase (SerRS) attaches serine (Ser) to tRNA^Sec^. Then, Ser-tRNA^Sec^ is phosphorylated by *O*-phosphoseryl-tRNASec kinase (PSTK) at seryl group to generate phosphoseryl—tRNASec (P-Ser-tRNA^Sec^). Finally, Sep(*O*-phosphoserine) tRNA:Sec [selenocysteine] tRNA synthase (SEPSECS) converts P-Ser-tRNA^Sec^ into selenocysteinyl (Sec)-tRNA^Sec^ with the participation of selenophosphate (SeP), which is the main Se donor in the process, produced by selenophosphate synthetase 2 (SEPHS2). Synthesized Sec is then incorporated into GPX4, which inhibits the lipid peroxidation and blocks ferroptosis. Selenoprotein P (SelP) transports Se across the membrane. The increased Se upregulates the expression of GPX4 through transcriptional coactivators, TRAP2C and Sp1.

Se deficiency has been revealed that promotes necroptosis and leads to damage in multiple tissues (83–86). MicroRNAs (miRNAs) are actively involved in the crosstalk between Se deficiency and necroptosis (Figure 2A). Through microRNAome analysis, Yang et al., identified that Se deficiency upregulated miR-200a-5p, which promoted RIPK3-dependent necroptosis *via* accumulating ROS and activating of mitogen-activated protein kinase (MAPK) pathway in chicken cardiac tissue (87). Se deficiency also suppressed miR-130 and miR-29a-3p in pig brain tissue. Decreased miR-130 and miR-29a-3p elevated CYLD and TNFR1 respectively, which facilitated the initiation of necroptosis (84, 88). Se deficiency deteriorated LPS-induced necroptosis by upregulating miR-16-5p, which subsequently activated PI3K/AKT pathway in chicken tracheal epithelial cells (85). Se supplement otherwise abolished LPS-induced oxidative stress and necroptosis *via* downregulating miR-155 and restoring its targeted gene, tumor necrosis factor receptor-associated factor 3 (TRAF3) (86). Besides regulation of miRNAs, Se was also found that antagonized cadmium-induced renal necroptosis by directly activating PI3K/AKT pathway *via* inhibiting PTEN (89).

The antioxidant function of Se also contributes to against necroptosis. Se supplement attenuated lead (Pb)-induced necroptosis through restoring redox balance and blocking MAPK/NF-κB pathway in chicken lymphocytes (90). Meanwhile, Se deficiency increased ROS level and augmented necroptosis in human uterine smooth muscle cells and swine liver (91, 92). However, Se nanoparticles reversely promoted ROS-mediated necroptosis with *via* upregulating TNF and interferon regulatory factor 1 (IRF1) in prostate cancer cells (93, 94).

#### Selenium and ferroptosis

Lipid peroxidation is essential for execution of ferroptosis. Iron-based oxidation of PUFA promotes the formation of lipid pores on the plasma membrane. GPX4, as a lipid hydroperoxidase, is designated to maintain the endogenous redox balance, which prevents the oxidation of PUFA and ferroptosis. GPX4 belongs to selenoprotein family, which converts lipid hydroperoxides to corresponding alcohols with the assistance of GSH (48, 49). As the active site, Sec incorporation is critical for GPX4 activity, which can be inhibited by RSL3 (52). GPX4 deficiency leads to accumulation of phospholipid hydroperoxides (PLOOH) and other lipid free radicals, which can be rapidly amplified by Fenton chain reaction and initiate ferroptosis (95).

As a selenoprotein, the expression and activity of GPX4 are regulated by Se (Figure 2B). Se supplement was shown to promote GPX4 expression in follicular helper T cells, which alleviated the accumulation of lipid peroxides and protected cells against ferroptotic cell death (96). Se protected neurons from I/R-induced ferroptosis *via* boosting GPX expression and activity (97). Alim et al. also observed that Se supplement increased the expression of selenoproteins, including GPX4, TXNRD1, GPX3, and selenoprotein P in neurons of intracerebral hemorrhage (ICH) mouse model (98). Se-mediated GPX4 upregulation was achieved *via* activation of transcriptional co-activators, TRAP2c and Sp1, which antagonized lipid oxidation, protected neurons, and improved brain functional recovery (98). Heat-stress-induced ferroptosis-like cell death was attenuated by Se-mediated upregulation of GPX4 and SOD activities in goat mammary epithelial cells (99). Bioinformatics analysis also suggested that lower Se level may be correlated with a disrupted iron metabolism and resultant ferroptosis in non-alcoholic fatty liver disease (NAFLD) liver (100). In cancer cells, Se is needed to promote cell survival by antagonizing ferroptosis. Vande Voorde et al. revealed that Se supplement alone was sufficient to promote survival and colony formation of breast cancer cells that proliferated at low density (101). Enhanced Sec-GPX4 axis restored the redox balance, which counteracted the ferroptosis (101). In addition, Se supplement also prevented spontaneous ferroptosis by increasing GPX4 expression in cultured adrenocortical carcinoma cells, which suggests that the ferroptotic sensitivity is relied on Se (102). Some chemicals can enhance Se sensitivity of GPX4. Fan et al. demonstrated that wedelolactone enhanced the transcription and Se sensitivity of GPX4, which attenuated oxidative stress-mediated ferroptosis and pyroptosis in chemical-induced acute pancreatitis (103). Curculigoside increased Se sensitivity and GPX4 transcription, which alleviated ferroptosis and associated oxidative stress in intestinal epithelial cells (104).

In addition to promote the expression of GPX4 and other selenoproteins, Se-based Sec insertion is required for the antioxidant function of GPX4 (105). Targeted mutation of Sec to Cys in GPX4 sensitized cells to peroxide-induced ferroptosis, accompanied with intracellular overoxidation (105). Another mutation, *Gpx4-U46S*, facilitated incorporation of Ser, instead of Sec, into the active site of GPX4, which impaired male spermatogenesis in heterozygous mice and were lethal at embryonic stage in homozygous mice (106). Liu et al. also observed a point mutation of GPX4, R152H damaged antioxidant activity and degradation of GPX4 (107).

The intracellular level of Se determines the Sec biosynthesis and expression of selenoproteins. The factors that regulate Se level also affect ferroptotic process. The cystine/glutamate antiporter, xCT, contributes to the uptake of Se. Upregulation of xCT subunits, solute carrier family 7 number 11 (SLCA11) or solute carrier family 3 member 2 (SLC3A2), increased the GPX4 expression, but paradoxically hypersensitized breast cancer cells to erastin or RSL3-induced ferroptosis (108). Similar observation was also reported by Carlisle et al. They showed that upregulation of SLC7A11 increased the uptake of Se, which contributed to selenophilic feature of cancer cells *via* promoting the biosynthesis of Sec and selenoproteins, against ferroptosis (109). Meanwhile, SEPHS2 was upregulated and detoxified the poisonous intermediate, selenide during Sec biosynthesis, which also promoted cancer cell survival (109). Selenoprotein P, a plasma membrane associated Se transporter, maintains the level of cellular selenoprotein. Deletion of selenoprotein P decreased GPX4 and selenoprotein K, and triggered ferroptosis-like cell death in pancreatic β cells, which was reversed by addition of Se (110). In addition, diaphanous-related formin-3 (DIAPH3) also upregulated cellular Se level and selenoprotein, TXNRD1, in pancreatic cancer cells (111). TXNRD1-mediated antioxidant effects enhanced malignancy of pancreatic cancer cells through antagonizing ferroptosis (111).

In contrast to prevent ferroptosis, selenite compound promotes ferroptosis. Sodium selenite triggered ferroptosis through downregulating SLC7A11, GSH and GPX4, but upregulating iron accumulation and lipid peroxidation in multiple cancer cells (112). Sodium selenite-induced ROS was scavenged by antioxidants such as SOD and Tiron, which antagonized resultant ferroptosis (112).

### Iron and regulated necrosis

Iron is an essential micronutrient, which plays vital functions in multiple biochemical activities, including oxidative metabolism, energy metabolism, and many catalytic reactions (113). Dietary iron appears in heme and non-heme forms, which is absorbed through apical surface of duodenal enterocytes and exported into the circulation (114). Hepcidin is the main regulator of plasma iron concentration. The binding of hepcidin facilitates the degradation of its receptor-ferroportin1, an iron efflux pump. The degradation blocks cellular iron export and downregulated serum iron level (115). In humans, iron deficiency may lead to anemia, infections and heart failure, while iron overload-caused oxidative damage also induces multiple diseases (116).

#### Iron and ferroptosis

**FIGURE 3:**
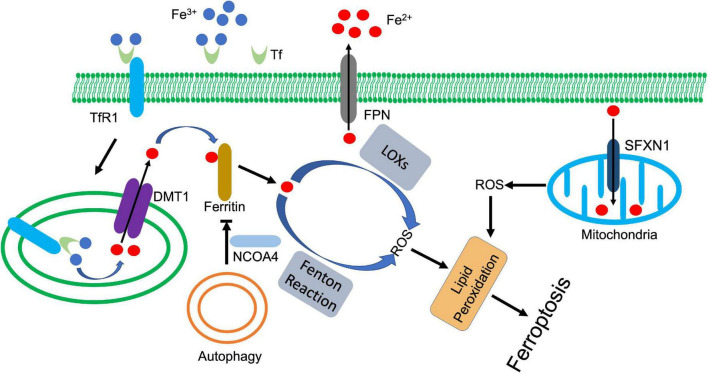
Iron metabolism and ferroptosis. Ferric state of iron (Fe^3+^) is bound to transferrin (Tf) in serum. Transferrin receptor 1 (TfR1) recognizes and binds the complex, which facilitates the endocytosis of ferric iron. The six-transmembrane epithelial antigen of the prostate-3 (Steap3) and acidic environment of the endosome jointly promote the reduction and release of ferrous iron (Fe^2+^). Divalent metal transporter 1 (DMT1) transports ferrous iron to cytoplasm, which is tightly controlled by ferritin. Autophagy (or ferritinophagy) selectively degrades ferritin with the coordination of nuclear receptor coactivator 4 (NCOA4), whose downregulation releases ferrous iron to activate lipid peroxidation, ultimately leading to ferroptosis. In addition, ferrous iron also triggers mitochondria-derived ROS through siderofexin (SFXN1). Ferroportin1 (FPN) can transport ferrous iron out of cell, which decreases the intracellular concentration of ferrous iron.

As we mentioned previously, aberrant accumulation of lipid peroxides, together with incapacity to eliminate them, leads to rapid membrane rupture and subsequent ferroptosis. Direct or indirect inactivation of GPX4 breaks the balance of lipid peroxide production and dissipation (117). Although it is still elusive, two primary mechanisms are found that contribute to lipid peroxidation in cells, which include iron-catalyzed autoxidation, and iron-dependent LOX-mediated lipid peroxidation (118–120). Both processes require the participation of iron (Figure 3). The iron-dependent non-enzymatic lipid peroxidation requires the generation of hydroxyl radical, which is catalyzed by Fenton reaction (Fe^2+^ + H_2_O_2_ → Fe^3+^ + HO⋅ + HO^–^) (118). The highly reactive hydroxyl radicals attack the labile bis-allylic hydrogen atom of lipids with PUFA tail, the products of which mediate the execution of ferroptosis (117, 118). On the other hand, LOX-derived lipid peroxidation also triggers ferroptosis. 15-LOX directly oxidized arachidonic acid and adrenoyl-phosphatidylethanolamines (PEs), which facilitated the execution of ferroptosis (121). 15-LOX and LOXE3 also mediated erastin-induced ferroptosis (52). GSH deficiency activated 12-LOX, which promoted lipid peroxidation and led to neuronal cell death (122). GPX4 inactivation led to 12/15 LOXs-dependent lipid peroxidation, which triggered apoptosis-inducing factor (AIF)-mediated cell death (123). Cytochrome P450 oxidoreductase (POR)-enabled membrane lipid peroxide overproduction promoted ferroptosis in cancer cells under the ferroptotic inducer treatment (124).

Due to its high redox activity, the iron transportation and delivery are under constant control with specific proteins. Extracellularly, transferrin binds ferric iron and promotes its endocytosis by interacting with transferrin receptor 1 on the cell surface (125). The acidic environment of endosome facilitates releases of iron from transferrin, which is then reduced by ferric reductases (Six-transmembrane epithelial antigen of protein 3, STEAP3) and transported to cytoplasm by divalent metal transporter 1 (DMT1) (126). The cytosolic ferrous iron is closely watched by a pool of molecules that constrain its prooxidative activity, and eventually deliver iron to its appropriate targets. These molecules consist of small molecules, such as GSH and macromolecules, such as PCBP and ferritin (127–130). These factors not only involve in iron delivery, but also engage in regulation of ferroptosis *via* modulating iron-mediated lipid peroxidation.

Ferritin, as iron storage proteins, is one of the most investigated regulators among these factors. Three forms of ferritin are known: ferritin heavy chain (FTH), ferritin light chain (FTL), and mitochondrial ferritin (FTMT) (131, 132). Degradation of ferritin increased cellular iron level, leading to accumulation of ROS and ultimately cell death (133). FTH deficiency downregulated ferroptotic regulator SLC7A11, which reduced GSH and increased lipid peroxidation, and ultimately promoted ferroptosis in cardiomyocytes (134). The protective role of FTMT against ferroptosis has also been detected in a series of investigations (135, 136). FTMT ablation promoted lipid peroxidation and ferroptosis, which deteriorated I/R-induced brain damage. Meanwhile, FTMT ablation potentiated hepcidin-mediated downregulation of ferroportin1, which further increased cellular iron level and promoted ferroptosis (136). In contrast, overexpression of FTMT reduced osteoblastic ferroptosis under high glucose condition (137).

Autophagy-mediated degradation of ferritin or ferritinophagy enhances intracellular iron level and ferroptosis. Erastin-triggered ROS activated autophagy in fibroblasts, which enhanced cellular iron level and promoted ferroptosis through degradation of ferritin and upregulation of transferrin receptor 1 (133). Nuclear receptor coactivator 4 (NCOA4) is an autophagic receptor that mediates the selective degradation of ferritin (138). Inhibition of NCOA4-mediated ferritinophagy antagonizes ferroptosis in various cells (138–140). However, ferritin was also found that can be directly degraded by lysosome in an autophagy-independent manner in cancer cells after artemisinin treatment (141). The released iron further upregulated the expression of siderofexin (SFXN1) on mitochondrial membrane, which transmitted cytoplasmic Fe^2+^ to mitochondria, leading to ROS production and ferroptosis (142). Besides autophagy, other proteins are also found involved in regulation of ferritin. Nuclear factor erythroid 2-related factor 2 (NRF2) upregulated the transcription of ferritin, which inhibits ferroptosis in various cells (143, 144). During the process, AMPK activated NRF2 (144), while p62 prevented NRF2 degradation and promoted its nuclear translocation (143). miR-335 suppressed FTH expression, exacerbating the neuronal ferroptosis in Parkinson’s disease (145). Prominin2, a pentaspanin protein that is implicated in regulation of lipid dynamics, promoted the formation of ferritin-containing multivesicular bodies and exosomes, which exported iron to inhibit ferroptosis (146).

Transferrin, together with transferrin receptor 1, are responsible for the cellular iron uptake that transport serum iron across the plasma membrane. Transferrin transcripts are rarely detected, except in liver and some cell types in brain. When serum transferrin level decreases, accumulated non-transferrin-bound iron induces damage to tissues, such as liver (147). Yu et al. revealed that transferrin knockout increased the sensitivity of hepatocytes to iron overload-induced ferroptosis and led to liver damage (148). Not limited to hepatocytes, transferrin knockdown also impaired tumorigenic capacity of melanoma cells by inducing ferroptosis, while they became more resistant to drug-induced ferroptosis when transferrin was aberrantly overexpressed (149). In addition, Gao et al. also demonstrated that transferrin itself was an inducer of ferroptosis. Endocytosis of iron-loaded transferrin that mediated by transferrin receptor 1, triggered ferroptosis in MEFs when the cells were upon amino acid starvation (150).

Transferrin receptor 1 regulates the intracellular iron concentration, which is closely correlated with iron-dependent ferroptosis. As an iron sensor, iron-responsive element-binding protein (IREB2) can stabilize transcripts of transferrin receptor 1, thereby increase intracellular iron concentration. Song et al. identified that OTUD1, a deubiquitinase, deubiquitinated and stabilized IREB2, which sequentially promoted transferrin receptor 1-mediated iron endocytosis and led to ROS-dependent ferroptosis (151). Another deubiquitinase, USP7, upregulated transferrin receptor 1 through stabilizing p53 in cardiomyocytes upon I/R injury. The activated USP7/p53/transferrin receptor 1 axis increased iron content and lipid peroxidation, which promoted ferroptosis (152). On the other hand, HUWE1, an E3 ligase negatively regulated ferroptosis *via* specifically targeting transferrin receptor 1 for proteasomal degradation. Suppression of HUWE1 remarkably enhanced sensitivity of hepatocytes to ferroptosis upon acute liver injury (153).

Ferroportin1 also regulates iron accumulation and associated ferroptosis alone or cooperatively. Ferroportin1 deficiency triggered ferroptosis in neurons, which contributed to multiple neurodegenerative diseases, such as Alzheimer’s disease (AD) and Incidence of ICH (154, 155). A decreased ferroportin1 deteriorates the transferrin receptor 1-mediated ferroptosis by accumulating more iron in ovarian cancer tumor-initiating cells, which made the cells exquisitely sensitive to ferroptotic agents (156).

Besides the factors described above, some other proteins also regulate ferroptosis through modulating iron metabolism. Iron regulatory protein 1 (IRP1) upregulated transferrin receptor 1 and downregulated ferroportin1 and ferritin, which facilitated erastin and RSL3-induced ferroptosis in melanoma cells (157). Yes-associated protein (YAP) is another critical regulator for ferroptosis. As a transcriptional stimulator, YAP promoted ferroptosis by upregulating several ferroptotic modulator including transferrin receptor 1 and acyl-CoA synthetase long chain family member 4 (ACSL4) (158). On the other hand, YAP stimulated the transcription of FTH and FTL with coordination of transcription factor CP2 (TFCP2). Inhibition of YAP or disturbed interaction between YAP and TFCP2 downregulated ferritin, which triggered ferroptosis by boosting labile iron (159, 160). The kinome screen revealed the important role of ATM in regulation of ferroptosis *via* modulating iron regulators. ATM inhibition enhanced the nuclear translocation of metal-regulatory transcription factor 1 (MTF1), which upregulated the expression of ferritins and ferroportin1. The coordinated changes decreased cellular labile iron and prevented iron-dependent ferroptosis in breast cancer cells upon cystine deprivation and erastin treatment (161). Glutaredoxin 5 (GLRX5) is a protein that transfers mitochondrial iron-sulfur cluster (ISC) to IRP, m-aconitase, and ferrochelatase (162), which counteracted ferroptosis. GLRX5 silencing boosted up iron-starvation response upon sulfasalazine treatment, which increased transferrin receptor 1, but decreased ferroportin1 and ferritin in head and neck cancer cells. The dysregulation of iron metabolism factors enhanced the sensitivity to induced ferroptosis (163).

#### Iron and other regulated necrosis

Iron overload and resultant ROS also contribute to necroptosis and pyroptosis. Dai et al. described that iron triggered necroptosis in primary cortical neurons, which was rescued by Nec1 (164). Iron-overload also correlates with necroptotic retinal disease. Increased iron and saturated transferrin were found in vitreous and subretinal fluid of patients with retinal detachment (RD), which contributed to induction of necroptosis and poor visual recovery. Meanwhile, overexpression of transferrin decreased iron level and antagonized RD-induced necroptosis (165). Mechanistically, iron-overload was able to increase intracellular ROS and trigger mitochondrial permeability transition (MPT) pore opening, which contributed to RIPK1/RIPK3/MLKL axis-mediated necroptosis in osteoblastic cells (166). Heme, an iron bonded molecule, also participates in the process of necroptosis. Free extracellular heme promoted TNFα production and resultant necroptosis *via* activation of Toll-like receptor 4 (TLR4) (167). The endocytosis heme was degraded by heme oxygenase-1 (HO-1) and released labile iron, which in turn generated ROS and induced necroptosis in macrophages (168).

Induction of pyroptosis by iron-mediated ROS has also been observed. Iron-activated ROS promoted oxidation and oligomerization of mitochondrial outer membrane protein Tom20 in melanoma cells. Oxidized Tom20 recruited Bax to mitochondria, which facilitated cytochrome c release and activated caspase-3. Then, activated caspase-3 cleaved GSDME and triggered pyroptotic cell death (169). Further study also demonstrated that iron supplement potentiated the anti-tumor effect of clinical ROS-inducing drugs through GSDME-dependent pyroptosis in xenografted melanoma mouse model (169).

### Zinc and regulated necrosis

Zinc (Zn) is an essential trace element, required for living organisms and biological processes. As a co-factor, Zn contributes to the catalytic activity of more than 300 enzymes that are actively involved in different levels of cellular signaling pathways including cell survival, proliferation, and differentiation (170). A proteome bioinformatics study even revealed that 10% human proteins were Zn-bonded and majority of them participated in the regulation of gene expression (171). Zn is absorbed by small intestine and excreted through intestine and pancreas (170, 172). Maintaining the homeostasis pf intracellular Zn ensures its binding proteins to function properly. Due to the inefficiency of Zn storage, it is vital for body to uptake this element consistently from daily diet (173), whose deficiency may lead to impairments of immune function, cell growth, cognitive function, and metabolism (174, 175).

#### Zn and Zn binding protein promote regulated necrosis

**FIGURE 4:**
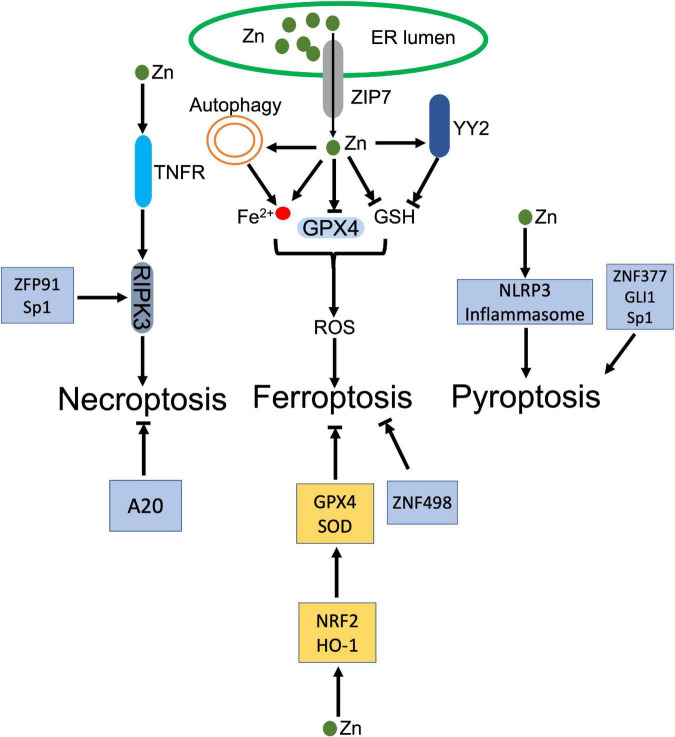
Dual roles of zinc (Zn) in regulated necrosis. ZIP transports Zn from ER to cytosol, which increases Zn level in cytoplasm. The increased Zn inhibits GPX4 and its cofactor GSH, and disturbs the intracellular iron homeostasis, which jointly trigger the accumulation of ROS and lead to ferroptosis. In addition, Zinc finger protein, YY2 inhibits GSH, which also activates ferroptosis. Besides ferroptosis, Zn and zinc finger proteins, ZFP91 and Sp1, facilitate activation of RIPK3 dependent necroptosis. Meanwhile, Zn activates NLRP3 inflammasome. Zinc finger proteins, ZNF377, GLI1, and Sp1 also induce pyroptosis. In the contrary, Zn upregulates GPX4 and GSH through NRF2 and HO-1 to counteract ferroptosis. ZNF498 and A20 also antagonize ferroptosis and necroptosis, respectively.

Zn and its binding proteins participate in the process of regulated necrosis positively and negatively (Figure 4). As an element, Zn is reported to promote regulated necrosis. High-leveled Zn exposure induced ferroptosis in non-small cell lung cancer (NSCLC) cells (176). Similar ferroptotic cell death was also observed in Zn-treated breast and renal cancer cells, which was rescued by Zn chelator, but not Iron chelator (177). Impaired redox balance contributes Zn compound-triggered ferroptosis. The execution of ferroptosis was attributed to elevation of ROS and lipid peroxidation, along with depletion of GSH and GPX4, and disturbance of iron homeostasis (178, 179). Genetic RNAi screen identified that ZIP7, a protein controls Zn transport from ER to cytosol, mediated Zn-induced ferroptosis. Inhibition of ZIP7 induced ER stress and prevented ferroptosis, whereas this protection was abolished by Zn supplement or depletion of ER stress associated protein, HERPUD1 (177). Autophagy may also contribute to Zn-induced ferroptosis. Qin et al. demonstrated that ZnONP-induced mitochondrial ROS activated AMPK-ULK1 axis, which triggered autophagic receptor, NCOA4-mediated ferritinophagy in endothelial cells (180). Disturbance of ferritinophagy profoundly mitigated ZnONP-induced ferroptosis (180). In addition, ZnONP was also found that selectively activated JNK pathways, which led to increased lipid peroxidation and ferroptotic cell death in neurons (181).

As the most abundant Zn binding proteins, zinc-finger proteins (ZFPs) involve in modulation of ferroptosis. Yin Yang 2 (YY2) protein manipulated cellular redox homeostasis, which induced tumor cell ferroptosis and suppressed tumorigenesis. Mechanistically, YY2 competed against its homolog YY1 to bind SLC7A11 promoter, which blocked transcription of SLC7A11 and associated GSH biosynthesis (182). Mutation of YY2 zinc-finger domains disturbed the interaction between YY2 and SLC7A11, and abrogated ferroptosis (182). A20 was identified that directly interacted with ACSL4, whose upregulation promoted erastin-induced ferroptosis in endothelial cells (183). MicroRNA, miR-17-92 targeted the A20-ACSL4 axis and prevented ferroptosis (183).

Besides ferroptosis, long-term exposure of Zn gluconate (ZG) was demonstrated that increased NLRP3 and IL-1β in olfactory neurons, which indicated activation of inflammasome and associated pyroptosis (184). Co-exposure of UVB and ZnONPs increased cytosolic and mitochondrial ROS, which triggered NLRP3 inflammasome activation and pyroptosis, accompanying with reduced autophagy and associated exosome release in keratinocytes (185). Zinc finger protein, ZNF377 was frequently silenced in multiple cancer cells, whose restoration promoted pyroptosis *via* increment of oxidative stress and ER stress (186). Glioma-associated oncogene family zinc finger 1 (GLI1) was upregulated in response to hypoxia exposure, which promoted pyroptosis in pulmonary artery smooth cells by enhancing the transcription of ASC, leading to pulmonary hypertension (187). Sp1, a zinc-finger transcription factor, was identified to promote transcription of zinc finger antisense 1 (ZFAS1), which enhanced LPS-induced pyroptosis and inhibited autophagy in sepsis-induced cardiac dysfunction (188).

Zn transporter, ZIP7 promoted necroptosis, whose knockout decreased the sensitivity of leukemia cells to necroptosis through impairing TNFR trafficking (189). Zinc finger protein 91 (ZFP91) was reported that stabilized RIPK1 through promoting RIPK1’s de-ubiquitination, thereby stabilizing RIPK1-RIPK3 interaction and facilitating necroptosis (190). Meanwhile, ZFP91 also contributed to the production of mitochondrial ROS, whose accumulation promoted TNF-mediated RIPK3-independent necroptosis (190). Sp1 directly regulated RIPK3 expression in cancer cells. Sp1 knockdown significantly decreased the transcription of RIPK3, thereby inhibited necroptosis, which was restored by re-expression of Sp1 (191).

#### Zn and Zn binding protein inhibit regulated necrosis

Souffriau et al. identified that Zn antagonized TNF-induced necroptosis in Paneth cells *via* modulating intestinal microbiota. Modified composition of the gut microbiota strongly downregulated interferon-stimulated response (ISRE) genes and IRF genes, ameliorating TNF-induced necroptosis (192). Zn also inhibited neuronal ferroptosis in spinal cord injured mouse model. Zn supplement rescued injured mitochondria and eliminated the accumulation of lipid peroxides through enhancing the expression of antioxidant enzymes, GPX4 and SOD, in a NRF2 and HO-1 dependent manner (193).

Zinc finger protein, A20 inhibited macrophage necroptosis, which prevented inflammatory arthritis in mice. The anti-inflammatory function depended on A20 zinc finger 7 (ZnF7) ubiquitin binding domain, whose mutation damaged A20-dependent inhibition of necroptosis and its anti-inflammatory function *in vivo* (194). In hepatocellular carcinoma, highly expression of ZNF498 deactivated p53-dependent transcription through blocking PKCδ- and p53INP1-mediated p53 phosphorylation at Ser46, which attenuated ferroptosis (195). Zinc Finger E-Box Binding Homeobox 2 (ZEB2) promoted activation of astrocytes or astrogliosis, which protected neurons by alleviating pyroptosis in ischemia-caused central nervous system injury (196).

### Vitamin E and regulated necrosis

Vitamin E family belongs to fat-soluble vitamins, consisting of four tocopherols (α-, β-, γ-, δ-tocopherol) and four tocotrienols (α-, β-, γ-, δ-tocopherol), which are well-known as lipophilic antioxidants (197). Accumulated investigations reveal their important roles in scavenging ROS and suppressing pro-inflammatory signaling (197, 198). Among these natural forms of vitamin E, α-tocopherol (α-Toc) is the predominant one that eliminates cellular lipid peroxides *via* trapping peroxyl radicals, which prevents ROS related cell death (198, 199).

**FIGURE 5:**
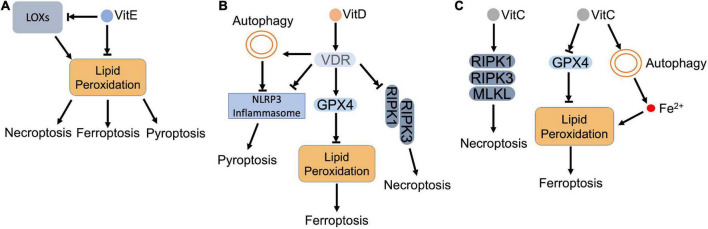
Vitamins and regulated necrosis. **(A)** Vitamin E. Vitamin E (VitE) attenuates regulated necrosis through downregulating the intracellular ROS. As lipophilic antioxidants, VitE can directly remove the lipid peroxides that accumulate in the cell. Meanwhile, VitE inhibits the activity of LOXs, which also blocks the lipid peroxidation. **(B)** Vitamin D. Vitamin D (VitD) activates vitamin D receptor (VDR), which (i) inhibits the formation of necrosome to block necroptosis; (ii) upregulates the expression of GPX4 to inhibit lipid peroxidation and resultant ferroptosis; (iii) inactivates inflammasome directly or *via* autophagy to inhibit pyroptosis. **(C)** Vitamin C. Vitamin C (VitC) upregulates the expression of RIPK1, RIPK3, and MLKL, which triggers necroptosis. In addition, VitC triggers lipid peroxidation by (i) inactivating GPX4: (ii) increasing the intracellular ferrous iron. The accumulated lipid peroxides lead to ferroptosis.

As an antioxidant, vitamin E was found that cooperated with GPX4 to remove lipid hydroperoxides in cells and animal models (200, 201; Figure 5A). Dietary supplement of vitamin E alleviated GPX4 deficiency-caused oxidative damage in endothelial cells, hepatocytes, T cells, myeloid cells, reticulocytes and neurons (200–205). Lipid peroxidation initiates the execution of ferroptosis. Vitamin E was able to eliminate lipid peroxides that were generated due to GPX4 depletion, which prevented ferroptosis in cultured hematopoietic stem and progenitor cells (206). In neurons, vitamin E supplement also attenuated GPX4 inhibition-mediated ferroptosis, which rescued neurons and improved the cognitive function and mortality (207, 208). Besides cytosolic GPX4, vitamin E also compensated mitochondrial GPX4 (mtGPX4) dysfunction-caused lipid peroxidation. Azuma et al. demonstrated that vitamin E blocked peroxided PE esterified with docosahexaenoic acid and rescued photoreceptor loss in retina of mtGPX4 knockout mice (209).

Vitamin E was also shown to antagonize ferroptosis through modulating LOX-mediated lipid peroxidation (210). Vitamin E can inhibit LOX activity by either downregulating its expression or competing for its substrate-binding site (199). Kagan et al. demonstrated that LOX-mediated PE oxidation in ER-associated compartment triggered ferroptosis as death signals in GPX4 deficient MEFs. Through suppressing LOX activity, vitamin E (tocopherols and tocotrienols) downregulated oxygenated PE species, preventing against ferroptosis (121). Systematical investigation revealed that α-tocotrienol and its metabolite, α-tocotrienol hydroquinone, inactivated 15-LOX by switching the enzyme’s non-heme iron from Fe^3+^ to Fe^2+^, which inhibited ferroptosis (211). In addition, vitamin E also downregulated LOX, but upregulated GPX4 in neurons of epileptic rat model. The restored redox balance attenuated neuronal ferroptosis and improved epileptic conditions (212).

Besides modulating these two enzymes, vitamin E also prevents ferroptosis that is induced directly by iron. Transferrin is responsible to restrain the intracellular iron pool, whose knockout promoted iron-dependent lipid peroxidation and ferroptosis, which weakened tumorigenic capacity of circulating melanoma cells in the bloodstream (149). Hong et al. observed that vitamin E was able to restore the resistance of tumor cells to ferroptotic inducers upon transferrin knockout, which promoted tumorigenesis of melanoma cells (149). Poly rC binding protein 1 (PCBP1) is a cytosolic iron chaperone that binds and transfers iron in cells. Deletion of PCBP1 releases labile iron, which leads to mitochondrial damage, lipid peroxidation and ferroptosis. Vitamin E, alone or together with coenzyme Q, removed iron-dependent oxidative stress and prevented ferroptosis in hepatocytes (213, 214).

In addition to ferroptosis, vitamin E also counteracts other types of regulated necrosis as an antioxidant. Kang et al. showed that lipid peroxidation facilitated caspase-11-dependent GSDMD cleavage and pyroptosis, while administration of vitamin E attenuated lipid peroxidation and resultant pyroptosis in myeloid lineage cells (215). Pretreatment of vitamin E also effectively reduced hydrogen peroxide-induced ROS and protected hepatocytes against pyroptosis (216). For necroptosis, vitamin E destabilized necrosome formation by attenuating ROS, which inhibited SMAC mimetic/TNFα-induced necroptosis in cancer cells (217). Basit et al. also reported that vitamin E ameliorated MPT pore opening and associated intracellular ROS, which blocked necroptosis and ferroptosis in melanoma cells (218).

### Vitamin D and regulated necrosis

Vitamin D is also a group of fat-soluble vitamins that contain two forms: vitamin D2 (ergocalciferol) and D3 (cholecalciferol) (219). In addition to oral ingestion from diary supplement, vitamin D can also be synthesized by skin after sun exposure (220). It has been long accepted that the primary role of vitamin D is to promote absorption and metabolism of calcium and phosphate, which maintain bone and muscle health. Recent progress also find that vitamin D deficiency may increase the risk of cancer, autoimmune disease, diabetes, and cardiovascular disease (219). Mechanistically, vitamin D can bind and activate vitamin D receptor (VDR) to regulate gene transcription and signal transduction in virtually every tissue (221).

Accumulated data reveal that vitamin D antagonizes regulated necrosis (Figure 5B). Vitamin D supplement ameliorated mitochondrial damage, enhanced total GPX activity, and reduced cellular iron level, which jointly prevented lipid peroxidation and ferroptosis in zebra fish liver (222). Hu et al. demonstrated that GPX4 was a target gene of VDR. Activated VDR stimulated GPX4 transcription, which removed lipid peroxides and prevented cisplatin-induced ferroptosis in kidney (223). Moreover, vitamin D also decreased Hamp1 (hepcidin-1 precursor) by modulating Keap1-NRF2-GPX4 and NF-κB-hepcidin axis. The decreased Hamp1 upregulated ferroportin1, which exported iron and prevented iron overload (222).

In addition to ferroptosis, serum vitamin D level was observed that negatively correlated with expression of necroptotic factors in IBD patients (224). Further study indicated that cytoplasmic translocation of VDR impeded the formation of necrosome, which suppressed necroptosis and ameliorated structural damage of intestinal epithelial cells in DSS-induced colitis mice (224). Consistent with the observation, RIPK1 was found that formed a complex with VDR and retained VDR in cytoplasm in MEFs and ovarian cancer cells. The formed complex not only blocked transcriptional activity of VDR, but also disturbed the necrosome formation (225).

For pyroptosis, vitamin D deficiency was found that deteriorated renal function by increasing pyroptotic inflammation in acute kidney injury (AKI) patients (226). Vitamin D supplement, on the other hand, restored redox balance and inhibited GSDMD-mediated pyroptosis (226). During the process, VDR was activated to mediate the anti-pyroptotic function of vitamin D. Jiang et al. showed that activation of VDR downregulated the expression of key pyroptotic proteins, such as NLRP3, cleaved caspase-1 and GSDMD, and inflammatory cytokines in cisplatin-injured kidney. Meanwhile, VDR activation inhibited NF-κB signaling *via* upregulating IκB, which also suggests that vitamin D/VDR alleviates pyroptosis *via* inhibiting NF-κB pathway (227). Moreover, vitamin D was found that benefited high fat diet (HFD)-induced hepatic injury by diminishing lipid-mediated activation of NLRP3 inflammasome and pyroptosis (228). In oral squamous carcinoma cells, vitamin D relieved platinum-induced pyroptosis by diminishing caspase-3 dependent GSDME cleavage (229). In addition, vitamin D also attenuates pyroptosis *via* modulating autophagy. Vitamin D was identified that activated AMPK and inhibited mTOR subsequently. The mTOR inhibition rescued high-glucose-induced pancreatic β-cell pyroptosis through blocking the formation of NLRP3 inflammasome (230). Pi et al. reported that vitamin D decreased ROS and activated autophagy, which prevented pyroptosis that induced by hypoxia/reoxygenation (H/R) in trophoblasts (231).

### Vitamin C and regulated necrosis

Vitamin C is a water-soluble antioxidant, which efficiently scavenges ROS under various stress conditions (232). It has two states, reduced ascorbate and oxidized dehydro-ascorbate (DHA). Sodium dependent vitamin C transporter (SVCT) family and glucose transporter (GLUT) family facilitate the absorbance of vitamin C along the entire intestine (22). The ascorbate is the predominant form of vitamin C that exists in the body due to its high affinity to the transporters, which contributes to the antioxidant feature of vitamin C (22). For example, ascorbate reduced tocopheroxyl radical to tocopherol, which prevented the generation of lipid peroxides (233). However, autoxidation of ascorbate also paradoxically produces ascorbyl radical, which ultimately generates highly reactive and cytotoxic hydroxyl radicals and hydrogen peroxides though reacting with transition metals. To maintain the balance of pro- and antioxidant activities, ascorbate is strictly regulated through its transporters (234).

Based on these features, pharmacological concentration of ascorbate has been applied to eliminate cancer cells. The cytotoxicity of pharmacological ascorbate includes ROS production and associated lipid peroxidation, which lead to ferroptosis (Figure 5C). Wang et al. reported that ascorbate inactivated GPX4, which led to accumulation of iron-dependent lipid peroxides and triggered ferroptosis in thyroid cancer cells. Meanwhile, ascorbate also promoted ferritin degradation *via* ferritinophagy. The released iron further deteriorated lipid peroxidation and ferroptosis (235). In addition, vitamin C also coordinates with anti-cancer drugs that synergistically enhances ferroptosis in cancer cells. Ascorbate was shown that potentiated the anti-cancer capacity of sorafenib, an oncogenic kinase inhibitor, by enhancing ferroptosis in liver cancer cells (236). In combination with cetuximab, ascorbate potentiated the cytotoxicity and overwhelmed the acquired resistance of cetuximab by triggering iron-dependent ferroptosis in colorectal cancer cells (237).

In regard to necroptosis, ascorbate exerts dual roles. Ascorbate treatment significantly increased the expression of necroptotic factors, RIPK1, RIPK3, and MLKL, which induced ROS-independent necroptosis in neurons (238). On the contrary, ascorbate, together with *N*-acetylcysteine (NAC), removed ROS and maintained mitochondrial integrity, which prevented necroptosis in mesenchymal stem cells (239). For pyroptosis, Tian et al. found that ascorbate, in combination with arsenic trioxide, stimulated ROS and promoted inflammasome formation, leading to pyroptosis in leukemia (240).

Besides the vitamins mentioned above, vitamin B6 is also found that involves in regulation of regulated necrosis. Vitamin B6, a water-soluble vitamin, restored the iron homeostasis and promoted the expression of antioxidant enzymes by activating NRF2, which restrained LPS-induced ferroptosis in myocytes (241). Vitamin B6 was also found that induced GSDME dependent pyroptosis in macrophages, but switched to MLKL dependent necroptosis when caspase-8 was blocked (242).

## Crosstalk between prooxidant and antioxidant micronutrients in regulated necrosis process

**FIGURE 6:**
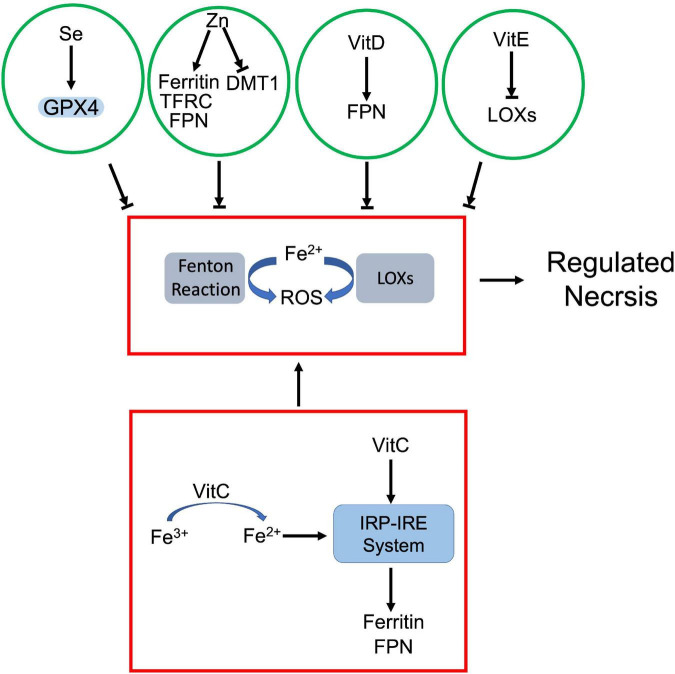
Crosstalk between prooxidant and antioxidant micronutrients in regulated necrosis process. Iron, as a prooxidant, catalyzes generation of ROS *via* Fenton reaction and LOXs. The antioxidant micronutrients counteract iron-mediated ROS production. Among them, selenium (Se) enhances GPX4 activity, and vitamin E (VitE) inhibits the activity of LOXs. Zinc (Zn) and vitamin D (VitD) attenuate iron-dependent ROS by modulating iron metabolism associated proteins. On the other hand, vitamin C (VitC) not only facilitates reduction of iron ion, but also upregulates iron metabolism associated proteins *via* IRP-IRE system, which jointly increase the cellular uptake of iron.

As a set of unstable molecules, cellular ROS are tightly controlled to ensure their appropriate functions. Once they overload, ROS will cause cellular damage and lead to cell death. Accumulated studies have demonstrated the critical role of ROS in signaling pathways of regulated necrosis. Micronutrients are engaged in regulation of intracellular ROS status due to their respective prooxidant or antioxidant properties. Among the trace elements, iron, predominantly known as a prooxidant metal, catalyzes lipid oxidation. On the other hand, vitamin E, Se, and Zn are classified as antioxidant elements. Vitamin E directly halts lipid peroxidation, while Se and Zn prevent ROS formation indirectly (243). Vitamin C has a multiplicity of antioxidant and prooxidant effects (244, 245). Indeed, their crosstalk affects cellular ROS level and the resultant execution of regulated necrosis (Figure 6).

As a prooxidant metal, labile iron can directly catalyze free radical formation *via* Fenton reaction. Meanwhile, iron and iron derivatives can incorporate into ROS-producing enzymes as essential cofactors. Both effects contribute to ROS-dependent regulated necrosis. For instance, iron-activated ROS induced necroptosis and pyroptosis in various cells (166, 169). Iron itself or iron-containing enzymes are able to produce lipid peroxides, which lead to ferroptosis (130). Se, as an antioxidant, antagonizes iron-triggered ROS. Se dependent GPX4-GSH axis attenuates iron-mediated lipid peroxidation and associated ferroptosis. Moreover, Se downregulated iron level in the body. Chareonpong-Kawamoto et al. showed that Se deficiency increased iron concentration and induced iron-overload in both serum and tissues of rat (246). The correlation suggests a negative feedback between Se and iron.

Zn also has a marked impact on iron metabolism. Clinical studies revealed that serum Zn levels positively correlated with serum iron status (247, 248). However, Zn antagonizes iron accumulation in tissues. Mechanistically, Zn deficiency dysregulated the interaction between iron regulatory proteins (IRPs) and their corresponding iron responsive elements (IREs), which upregulated transferrin receptor 1 and ferritin, but downregulated DMT1 in adipocytes (249). Meanwhile, Zn supplement also induced ferroportin1 expression in zebra fish gills (250). It is established that iron is a prooxidant while Zn is an antioxidant due to their respective physicochemical properties (251). The negative correlation between Zn and iron further supports that Zn is able to neutralize iron-derived ROS.

Vitamins also regulate cellular iron uptake and metabolism. Among them, vitamin C is identified as the most important modulator of cellular iron metabolism, which affects iron uptake, storage, and efflux. Ascorbate is known not only to enhance non-heme iron absorption in gut, but also promotes transferrin or non-transferrin dependent cellular iron uptake (252, 253). As an electron donor, ascorbate facilitates the reduction of ferric ions to ferrous ions, which promotes non-transferrin-bound iron cellular uptake and cytoplasmic release of transferrin-bound iron from endosome (253). Besides iron uptake, ascorbate also modulates iron homeostasis through altering the expression of key iron metabolism associated proteins, such as ferritin and ferroportin1 (253). For instance, ascorbate increased ferritin expression through activating IRP-IRE system (254) or by inhibiting lysosomal ferritin degradation (255). However, ascorbate was also found that promoted ferritin degradation through ferritinophagy, which exaggerated iron-mediated lipid peroxidation and resultant ferroptosis (235). Moreover, ascorbate was able to block iron release in various cell types. Although it is still unclear whether this iron efflux is ferroportin1-dependent, ascorbate did increase ferroportin1 expression in intestinal epithelial-like cells (253).

Besides vitamin C, vitamin D was also found that upregulated ferroportin1 *via* downregulating hepcidin 1, which exported iron and prevented iron overload and iron-dependent ferroptosis (222). Vitamin E, as a potent oxygen radical scavenger, antagonizes iron-derived lipid peroxidation directly or indirectly. On one hand, vitamin E can directly reduce the intracellular iron-derived ROS (149). On the other hand, vitamin E downregulates LOX expression and inactivates their activity, which inhibits lipid peroxidation (121, 211). Both effects prevent iron-dependent ROS propagation and inhibit regulated necrosis.

## Conclusion

Regulated necrosis has become an attractive research field due to their causative role in multiple diseases. Pharmacological targeting their signaling pathways may provide alternative opportunities for the therapeutics by either triggering or preventing cell death in diseases. Such as in tumors, triggering of regulated necrosis has been proposed as a routine for effective targeted therapy to overwhelm the apoptotic resistance (256). On the other side, prevention of regulated necrosis may yield encouraging outcomes in diseases, such as IR injury and neurodegenerative disease (257). The recognition that micronutrients actively participate in regulation of regulated necrosis, opens a new avenue for the development of micronutrients as a novel adjuvant therapy. As essential components, micronutrients are involved in modulating metabolism as cofactors or coenzymes, or as antioxidant. Oxidative metabolism produced-ROS can be quenched by micronutrients or their related enzyme systems. Micronutrient deficiency-caused oxidative stress triggers regulated necrosis, which contributes to the etiology of multiple diseases. Appropriate manipulation of micronutrients and associated intracellular ROS benefit diseases by modulating regulated necrosis. A deeper understanding of the crosstalk between regulated necrosis and micronutrients will be instrumental in designing novel and effective therapeutics in order to treat diseases that involve cell death.

## Author contributions

LZ and DZ conceptualized and wrote the major part of the manuscript. JL, JW, ZD, MW, and RS reviewed and edited the manuscript. All authors contributed to the article and approved the submitted version.

## Glossary

ACSL4: acyl-CoA synthetase long chain family member 4
AIF: apoptosis inducing factor
AIM2: absent in melanoma 2
AKI: acute kidney injury
cIAP1/2: cellular inhibitor of apoptosis protein ½
DAMP: danger-associated molecular pattern
DHA: dehydro-ascorbate
DIAPH3: diaphanous-related formin-3
DMT1: divalent metal transporter 1
FADD: FAS-associated death domain
FTH: ferritin heavy chain
FTL: ferritin light chain
FTMT: mitochondrial ferritin
GLRX5: glutaredoxin 5
GLUT: glucose transporter
GSH: glutathione
GPX4: glutathione peroxidase 4
GSDMD: gasdermin D
HFD: high fat diet
HO-1: heme oxygenase-1
H/R: hypoxia/reoxygenation
IBD: inflammatory bowel disease
ICH: intracellular hemorrhage
IL-1β: interleukin-1β
IL-18: interleukin-18
I/R: ischemia/reperfusion
IRE: iron responsive element
IREB2: iron-responsive element-binding protein
IRF1: interferon regulatory factor 1
IRP: iron regulatory protein 1
ISC: iron-sulfur cluster
ISRE: interferon-stimulated response
LOX: lipoxygenase
LPS: lipopolysaccharide
LUBAC: linear ubiquitination (M1-Ubi) assembly complex
MAPK: mitogen-activated protein kinase
MEF: mouse embryonic fibroblast
miRNA: microRNA
MLKL: mixed-lineage kinase domain-like pseudokinase
MPT: mitochondrial permeability transition
MTF1: metal-regulatory transcription factor 1
NAFLD: non-alcoholic fatty liver disease
NAC: *N*-acetylcysteine
NCOA4: nuclear receptor coactivator 4
Nec-1: necrostatin-1
NLRC4 NLR: family caspase activation and recruitment domain (CARD) containing 4
NLRP1B: NLR family pyrin domain-containing 1B
NLRP3: NOD-like receptor pyrin domain-containing protein 3
NRF2: nuclear factor erythroid 2-related factor 2
PAMP: pathogen-associated molecular pattern
PCBP1: Poly rC binding protein 1
PCD: programmed cell death
PE: phosphatidylethanolamine
PRR: pattern recognition receptor
PUFA: polyunsaturated fatty acid
PLOOH: phospholipid hydroperoxides
RD: retinal detachment
RIPK1: receptor-interacting protein kinase 1
ROS: reactive oxygen species
RSL3: RAS-selective lethal 3
Se: selenium
Sec: selenocysteine
SECIS: Sec insertion sequence
SEPHS2: selenophosphate synthetase 2
SEPSECS: Sep(O-phosphoserine) tRNA:Sec [selenocysteine] tRNA synthase
SFXN1: siderofexin
SLCA11: solute carrier family 7 number 11
SPB SECIS: binding protein 2
SVCT: vitamin C transporter
TCR: T cell receptor
TFCP2: transcription factor CP2
TLR4: Toll-like receptor 4
TNFα: tumor necrosis factor α
TNFR: TNF receptor
TRADD: TNFR-associated death domain
TRAF2/5: TNFR-associated factor 2/5
TXNRD: thioredoxin reductases
VDR: vitamin D receptor
YAP: Yes-associated protein
YY2: Yin Yang 2
ZFAS1: zinc finger antisense 1
ZG: Zn gluconate
Zn: zinc
ZFP: zinc finger protein.

## References

[1] Riedl SJ Shi Y. Molecular mechanisms of caspase regulation during apoptosis. *Nat Rev Mol Cell Biol.* (2004) 5:897–907. 10.1038/nrm1496 15520809

[2] Meier P Finch A Evan G. Apoptosis in development. *Nature.* (2000) 407:796–801. 10.1038/35037734 11048731

[3] Favaloro B Allocati N Graziano V Di Ilio C De Laurenzi V. Role of apoptosis in disease. *Aging.* (2012) 4:330–49. 10.18632/aging.100459 22683550 PMC3384434

[4] Syntichaki P Tavernarakis N. Death by necrosis. Uncontrollable catastrophe, or is there order behind the chaos? *EMBO Rep.* (2002) 3:604–9. 10.1093/embo-reports/kvf138 12101090 PMC1084192

[5] Proskuryakov SY Konoplyannikov AG Gabai VL. Necrosis: a specific form of programmed cell death? *Exp Cell Res.* (2003) 283:1–16. 10.1016/s0014-482700027-7 12565815

[6] Vanlangenakker N Vanden Berghe T Krysko DV Festjens N Vandenabeele P. Molecular mechanisms and pathophysiology of necrotic cell death. *Curr Mol Med.* (2008) 8:207–20. 10.2174/156652408784221306 18473820

[7] Gunther C Neumann H Neurath MF Becker C. Apoptosis, necrosis and necroptosis: cell death regulation in the intestinal epithelium. *Gut.* (2013) 62:1062–71. 10.1136/gutjnl-2011-301364 22689519

[8] Linkermann A De Zen F Weinberg J Kunzendorf U Krautwald S. Programmed necrosis in acute kidney injury. *Nephrol Dial Transplant.* (2012) 27:3412–9. 10.1093/ndt/gfs373 22942173

[9] Krishna M. Patterns of necrosis in liver disease. *Clin Liver Dis.* (2017) 10:53–6. 10.1002/cld.653 30992760 PMC6467231

[10] Degterev A Huang Z Boyce M Li Y Jagtap P Mizushima N Chemical inhibitor of nonapoptotic cell death with therapeutic potential for ischemic brain injury. *Nat Chem Biol.* (2005) 1:112–9. 10.1038/nchembio711 16408008

[11] Dixon SJ Lemberg KM Lamprecht MR Skouta R Zaitsev EM Gleason CE Ferroptosis: an iron-dependent form of nonapoptotic cell death. *Cell.* (2012) 149:1060–72. 10.1016/j.cell.2012.03.042 22632970 PMC3367386

[12] Dixon SJ Stockwell BR. The hallmarks of ferroptosis. *Annu Rev Cancer Biol.* (2019) 3:35–54. 10.1146/annurev-cancerbio-030518-055844 PMC12851585 41613499

[13] Fang Y Tian S Pan Y Li W Wang Q Tang Y Pyroptosis: a new frontier in cancer. *Biomed Pharmacother.* (2020) 121:109595. 10.1016/j.biopha.2019.109595 31710896

[14] Bergsbaken T Fink SL Cookson BT. Pyroptosis: host cell death and inflammation. *Nat Rev Microbiol.* (2009) 7:99–109. 10.1038/nrmicro2070 19148178 PMC2910423

[15] Circu ML Aw TY. Reactive oxygen species, cellular redox systems, and apoptosis. *Free Radic Biol Med.* (2010) 48:749–62. 10.1016/j.freeradbiomed.2009.12.022 20045723 PMC2823977

[16] Zoidis E Seremelis I Kontopoulos N Danezis GP. Selenium-dependent antioxidant enzymes: actions and properties of selenoproteins. *Antioxidants (Basel).* (2018) 7:66. 10.3390/antiox7050066 29758013 PMC5981252

[17] Zhang Y Su SS Zhao S Yang Z Zhong CQ Chen X RIP1 autophosphorylation is promoted by mitochondrial ROS and is essential for RIP3 recruitment into necrosome. *Nat Commun.* (2017) 8:14329. 10.1038/ncomms14329 28176780 PMC5309790

[18] Yang WS Stockwell BR. Ferroptosis: death by lipid peroxidation. *Trends Cell Biol.* (2016) 26:165–76. 10.1016/j.tcb.2015.10.014 26653790 PMC4764384

[19] Wang Y Shi P Chen Q Huang Z Zou D Zhang J Mitochondrial ROS promote macrophage pyroptosis by inducing GSDMD oxidation. *J Mol Cell Biol.* (2019) 11:1069–82. 10.1093/jmcb/mjz020 30860577 PMC6934151

[20] Smith AD Panickar KS Urban JF Jr Dawson HD. Impact of micronutrients on the immune response of animals. *Annu Rev Anim Biosci.* (2018) 6:227–54. 10.1146/annurev-animal-022516-022914 29447473

[21] Shenkin A. Micronutrients in health and disease. *Postgrad Med J.* (2006) 82:559–67. 10.1136/pgmj.2006.047670 16954450 PMC2585731

[22] Szarka A Kapuy O Lorincz T Banhegyi G. Vitamin C and cell death. *Antioxid Redox Signal.* (2021) 34:831–44. 10.1089/ars.2019.7897 32586104

[23] Conrad M Proneth B. Selenium: tracing another essential element of ferroptotic cell death. *Cell Chem Biol.* (2020) 27:409–19. 10.1016/j.chembiol.2020.03.012 32275866

[24] Choi ME Price DR Ryter SW Choi AMK. Necroptosis: a crucial pathogenic mediator of human disease. *JCI Insight.* (2019) 4:e128834. 10.1172/jci.insight.128834 31391333 PMC6693822

[25] Orozco S Oberst A. RIPK3 in cell death and inflammation: the good, the bad, and the ugly. *Immunol Rev.* (2017) 277:102–12. 10.1111/imr.12536 28462521 PMC5419046

[26] Moujalled DM Cook WD Okamoto T Murphy J Lawlor KE Vince JE TNF can activate RIPK3 and cause programmed necrosis in the absence of RIPK1. *Cell Death Dis.* (2013) 4:e465. 10.1038/cddis.2012.201 23328672 PMC3563989

[27] Amarante-Mendes GP Adjemian S Branco LM Zanetti LC Weinlich R Bortoluci KR. Pattern recognition receptors and the host cell death molecular machinery. *Front Immunol.* (2018) 9:2379. 10.3389/fimmu.2018.02379 30459758 PMC6232773

[28] Osborn SL Diehl G Han SJ Xue L Kurd N Hsieh K Fas-associated death domain (FADD) is a negative regulator of T-cell receptor-mediated necroptosis. *Proc Natl Acad Sci U.S.A.* (2010) 107:13034–9. 10.1073/pnas.1005997107 20615958 PMC2919948

[29] Lalaoui N Lindqvist LM Sandow JJ Ekert PG. The molecular relationships between apoptosis, autophagy and necroptosis. *Semin Cell Dev Biol.* (2015) 39:63–9. 10.1016/j.semcdb.2015.02.003 25736836

[30] Kim HJ Hwang KE Park DS Oh SH Jun HY Yoon KH Shikonin-induced necroptosis is enhanced by the inhibition of autophagy in non-small cell lung cancer cells. *J Transl Med.* (2017) 15:123. 10.1186/s12967-017-1223-7 28569199 PMC5452303

[31] Huang CY Kuo WT Huang YC Lee TC Yu LC. Resistance to hypoxia-induced necroptosis is conferred by glycolytic pyruvate scavenging of mitochondrial superoxide in colorectal cancer cells. *Cell Death Dis.* (2013) 4:e622. 10.1038/cddis.2013.149 23640464 PMC3674358

[32] Micheau O Tschopp J. Induction of TNF receptor I-mediated apoptosis via two sequential signaling complexes. *Cell.* (2003) 114:181–90. 10.1016/s0092-867400521-x 12887920

[33] Vandenabeele P Galluzzi L Vanden Berghe T Kroemer G. Molecular mechanisms of necroptosis: an ordered cellular explosion. *Nat Rev Mol Cell Biol.* (2010) 11:700–14. 10.1038/nrm2970 20823910

[34] Bertrand MJ Milutinovic S Dickson KM Ho WC Boudreault A Durkin J cIAP1 and cIAP2 facilitate cancer cell survival by functioning as E3 ligases that promote RIP1 ubiquitination. *Mol Cell.* (2008) 30:689–700. 10.1016/j.molcel.2008.05.014 18570872

[35] Ea CK Deng L Xia ZP Pineda G Chen ZJ. Activation of IKK by TNFalpha requires site-specific ubiquitination of RIP1 and polyubiquitin binding by NEMO. *Mol Cell.* (2006) 22:245–57. 10.1016/j.molcel.2006.03.026 16603398

[36] Li H Kobayashi M Blonska M You Y Lin X. Ubiquitination of RIP is required for tumor necrosis factor alpha-induced NF-kappaB activation. *J Biol Chem.* (2006) 281:13636–43. 10.1074/jbc.M600620200 16543241

[37] Draber P Kupka S Reichert M Draberova H Lafont E de Miguel D LUBAC-recruited CYLD and A20 regulate gene activation and cell death by exerting opposing effects on linear ubiquitin in signaling complexes. *Cell Rep.* (2015) 13:2258–72. 10.1016/j.celrep.2015.11.009 26670046 PMC4688036

[38] Kovalenko A Chable-Bessia C Cantarella G Israel A Wallach D Courtois G. The tumour suppressor CYLD negatively regulates NF-kappaB signalling by deubiquitination. *Nature.* (2003) 424:801–5. 10.1038/nature01802 12917691

[39] Shan B Pan H Najafov A Yuan J. Necroptosis in development and diseases. *Genes Dev.* (2018) 32:327–40. 10.1101/gad.312561.118 29593066 PMC5900707

[40] Hitomi J Christofferson DE Ng A Yao J Degterev A Xavier RJ Identification of a molecular signaling network that regulates a cellular necrotic cell death pathway. *Cell.* (2008) 135:1311–23. 10.1016/j.cell.2008.10.044 19109899 PMC2621059

[41] Wei R Xu LW Liu J Li Y Zhang P Shan B SPATA2 regulates the activation of RIPK1 by modulating linear ubiquitination. *Genes Dev.* (2017) 31:1162–76. 10.1101/gad.299776.117 28701375 PMC5538438

[42] Dziedzic SA Su Z Jean Barrett V Najafov A Mookhtiar AK Amin P ABIN-1 regulates RIPK1 activation by linking Met1 ubiquitylation with Lys63 deubiquitylation in TNF-RSC. *Nat Cell Biol.* (2018) 20:58–68. 10.1038/s41556-017-0003-1 29203883 PMC5741489

[43] Newton K Wickliffe KE Dugger DL Maltzman A Roose-Girma M Dohse M Cleavage of RIPK1 by caspase-8 is crucial for limiting apoptosis and necroptosis. *Nature.* (2019) 574:428–31. 10.1038/s41586-019-1548-x 31511692

[44] Bohgaki T Mozo J Salmena L Matysiak-Zablocki E Bohgaki M Sanchez O Caspase-8 inactivation in T cells increases necroptosis and suppresses autoimmunity in Bim-/- mice. *J Cell Biol.* (2011) 195:277–91. 10.1083/jcb.201103053 22006951 PMC3198166

[45] Cho YS Challa S Moquin D Genga R Ray TD Guildford M Phosphorylation-driven assembly of the RIP1-RIP3 complex regulates programmed necrosis and virus-induced inflammation. *Cell.* (2009) 137:1112–23. 10.1016/j.cell.2009.05.037 19524513 PMC2727676

[46] Linkermann A Green DR. Necroptosis. *N Engl J Med.* (2014) 370:455–65. 10.1056/NEJMra1310050 24476434 PMC4035222

[47] Murphy JM Czabotar PE Hildebrand JM Lucet IS Zhang JG Alvarez-Diaz S The pseudokinase MLKL mediates necroptosis via a molecular switch mechanism. *Immunity.* (2013) 39:443–53. 10.1016/j.immuni.2013.06.018 24012422

[48] Jiang X Stockwell BR Conrad M. Ferroptosis: mechanisms, biology and role in disease. *Nat Rev Mol Cell Biol.* (2021) 22:266–82. 10.1038/s41580-020-00324-8 33495651 PMC8142022

[49] Pierzynowska K Rintz E Gaffke L Wegrzyn G. Ferroptosis and its modulation by autophagy in light of the pathogenesis of lysosomal storage diseases. *Cells.* (2021) 10:365. 10.3390/cells10020365 33578654 PMC7916399

[50] Lei P Bai T Sun Y. Mechanisms of ferroptosis and relations with regulated cell death: a review. *Front Physiol.* (2019) 10:139. 10.3389/fphys.2019.00139 30863316 PMC6399426

[51] Lu SC. Glutathione synthesis. *Biochim Biophys Acta.* (2013) 1830:3143–53. 10.1016/j.bbagen.2012.09.008 22995213 PMC3549305

[52] Yang WS Kim KJ Gaschler MM Patel M Shchepinov MS Stockwell BR. Peroxidation of polyunsaturated fatty acids by lipoxygenases drives ferroptosis. *Proc Natl Acad Sci U.S.A.* (2016) 113:E4966–75. 10.1073/pnas.1603244113 27506793 PMC5003261

[53] Zheng X Chen W Gong F Chen Y Chen E. The role and mechanism of pyroptosis and potential therapeutic targets in sepsis: a review. *Front Immunol.* (2021) 12:711939. 10.3389/fimmu.2021.711939 34305952 PMC8293747

[54] Man SM Karki R Kanneganti TD. Molecular mechanisms and functions of pyroptosis, inflammatory caspases and inflammasomes in infectious diseases. *Immunol Rev.* (2017) 277:61–75. 10.1111/imr.12534 28462526 PMC5416822

[55] Martinon F Tschopp J. Inflammatory caspases and inflammasomes: master switches of inflammation. *Cell Death Differ.* (2007) 14:10–22. 10.1038/sj.cdd.4402038 16977329

[56] Sborgi L Ruhl S Mulvihill E Pipercevic J Heilig R Stahlberg H GSDMD membrane pore formation constitutes the mechanism of pyroptotic cell death. *EMBO J.* (2016) 35:1766–78. 10.15252/embj.201694696 27418190 PMC5010048

[57] He WT Wan H Hu L Chen P Wang X Huang Z Gasdermin D is an executor of pyroptosis and required for interleukin-1beta secretion. *Cell Res.* (2015) 25:1285–98. 10.1038/cr.2015.139 26611636 PMC4670995

[58] Rathinam VA Fitzgerald KA. Inflammasome complexes: emerging mechanisms and effector functions. *Cell.* (2016) 165:792–800. 10.1016/j.cell.2016.03.046 27153493 PMC5503689

[59] Kayagaki N Stowe IB Lee BL O’Rourke K Anderson K Warming S Caspase-11 cleaves gasdermin D for non-canonical inflammasome signalling. *Nature.* (2015) 526:666–71. 10.1038/nature15541 26375259

[60] Shi J Zhao Y Wang K Shi X Wang Y Huang H Cleavage of GSDMD by inflammatory caspases determines pyroptotic cell death. *Nature.* (2015) 526:660–5. 10.1038/nature15514 26375003

[61] Sarhan J Liu BC Muendlein HI Li P Nilson R Tang AY Caspase-8 induces cleavage of gasdermin D to elicit pyroptosis during Yersinia infection. *Proc Natl Acad Sci U.S.A.* (2018) 115:E10888–97. 10.1073/pnas.1809548115 30381458 PMC6243247

[62] Orning P Weng D Starheim K Ratner D Best Z Lee B Pathogen blockade of TAK1 triggers caspase-8-dependent cleavage of gasdermin D and cell death. *Science.* (2018) 362:1064–9. 10.1126/science.aau2818 30361383 PMC6522129

[63] Wang Y Gao W Shi X Ding J Liu W He H Chemotherapy drugs induce pyroptosis through caspase-3 cleavage of a gasdermin. *Nature.* (2017) 547:99–103. 10.1038/nature22393 28459430

[64] Sanmartin C Plano D Palop JA. Selenium compounds and apoptotic modulation: a new perspective in cancer therapy. *Mini Rev Med Chem.* (2008) 8:1020–31. 10.2174/138955708785740625 18782054

[65] Labunskyy VM Hatfield DL Gladyshev VN. Selenoproteins: molecular pathways and physiological roles. *Physiol Rev.* (2014) 94:739–77. 10.1152/physrev.00039.2013 24987004 PMC4101630

[66] Sanmartin C Plano D Sharma AK Palop JA. Selenium compounds, apoptosis and other types of cell death: an overview for cancer therapy. *Int J Mol Sci.* (2012) 13:9649–72. 10.3390/ijms13089649 22949823 PMC3431821

[67] Chambers I Frampton J Goldfarb P Affara N McBain W Harrison PR. The structure of the mouse glutathione peroxidase gene: the selenocysteine in the active site is encoded by the ‘termination’ codon, TGA. *EMBO J.* (1986) 5:1221–7. 10.1002/j.1460-2075.1986.tb04350.x 3015592 PMC1166931

[68] Hatfield DL Tsuji PA Carlson BA Gladyshev VN. Selenium and selenocysteine: roles in cancer, health, and development. *Trends Biochem Sci.* (2014) 39:112–20. 10.1016/j.tibs.2013.12.007 24485058 PMC3943681

[69] Buettner C Harney JW Berry MJ. The *Caenorhabditis elegans* homologue of thioredoxin reductase contains a selenocysteine insertion sequence (SECIS) element that differs from mammalian SECIS elements but directs selenocysteine incorporation. *J Biol Chem.* (1999) 274:21598–602. 10.1074/jbc.274.31.21598 10419466

[70] Squires JE Berry MJ. Eukaryotic selenoprotein synthesis: mechanistic insight incorporating new factors and new functions for old factors. *IUBMB Life.* (2008) 60:232–5. 10.1002/iub.38 18344183

[71] de Jesus LA Hoffmann PR Michaud T Forry EP Small-Howard A Stillwell RJ Nuclear assembly of UGA decoding complexes on selenoprotein mRNAs: a mechanism for eluding nonsense-mediated decay? *Mol Cell Biol.* (2006) 26:1795–805. 10.1128/MCB.26.5.1795-1805.2006 16478999 PMC1430236

[72] Ganyc D Talbot S Konate F Jackson S Schanen B Cullen W Impact of trivalent arsenicals on selenoprotein synthesis. *Environ Health Perspect.* (2007) 115:346–53. 10.1289/ehp.9440 17431482 PMC1849912

[73] Arner ES. Selenoproteins-What unique properties can arise with selenocysteine in place of cysteine? *Exp Cell Res.* (2010) 316:1296–303. 10.1016/j.yexcr.2010.02.032 20206159

[74] Lobanov AV Hatfield DL Gladyshev VN. Eukaryotic selenoproteins and selenoproteomes. *Biochim Biophys Acta.* (2009) 1790:1424–8. 10.1016/j.bbagen.2009.05.014 19477234 PMC3471088

[75] Guillin OM Vindry C Ohlmann T Chavatte L. Selenium, selenoproteins and viral infection. *Nutrients.* (2019) 11:2101. 10.3390/nu11092101 31487871 PMC6769590

[76] Brown KM Arthur JR. Selenium, selenoproteins and human health: a review. *Public Health Nutr.* (2001) 4:593–9. 10.1079/phn2001143 11683552

[77] Nirgude S Choudhary B. Insights into the role of GPX3, a highly efficient plasma antioxidant, in cancer. *Biochem Pharmacol.* (2021) 184:114365. 10.1016/j.bcp.2020.114365 33310051

[78] Brigelius-Flohe R Kipp AP. Physiological functions of GPx2 and its role in inflammation-triggered carcinogenesis. *Ann N Y Acad Sci.* (2012) 1259:19–25. 10.1111/j.1749-6632.2012.06574.x 22758632

[79] Brigelius-Flohe R Maiorino M. Glutathione peroxidases. *Biochim Biophys Acta.* (2013) 1830:3289–303. 10.1016/j.bbagen.2012.11.020 23201771

[80] Dagnell M Schmidt EE Arner ESJ. The A to Z of modulated cell patterning by mammalian thioredoxin reductases. *Free Radic Biol Med.* (2018) 115:484–96. 10.1016/j.freeradbiomed.2017.12.029 29278740 PMC5771652

[81] Arner ES. Focus on mammalian thioredoxin reductases–important selenoproteins with versatile functions. *Biochim Biophys Acta.* (2009) 1790:495–526. 10.1016/j.bbagen.2009.01.014 19364476

[82] Patwardhan RS Sharma D Sandur SK. Thioredoxin reductase: an emerging pharmacologic target for radiosensitization of cancer. *Transl Oncol.* (2022) 17:101341. 10.1016/j.tranon.2022.101341 35078017 PMC8790659

[83] Zhang Y Zhang J Bao J Tang C Zhang Z. Selenium deficiency induced necroptosis, Th1/Th2 imbalance, and inflammatory responses in swine ileum. *J Cell Physiol.* (2021) 236:222–34. 10.1002/jcp.29836 32488864

[84] Cui J Liu H Xu S. Selenium-deficient diet induces necroptosis in the pig brain by activating TNFR1 via mir-29a-3p. *Metallomics.* (2020) 12:1290–301. 10.1039/d0mt00032a 32568328

[85] Wang L Shi X Zheng S Xu S. Selenium deficiency exacerbates LPS-induced necroptosis by regulating miR-16-5p targeting PI3K in chicken tracheal tissue. *Metallomics.* (2020) 12:562–71. 10.1039/c9mt00302a 32125337

[86] Zhirong Z Qiaojian Z Chunjing X Shengchen W Jiahe L Zhaoyi L Methionine selenium antagonizes LPS-induced necroptosis in the chicken liver via the miR-155/TRAF3/MAPK axis. *J Cell Physiol.* (2021) 236:4024–35. 10.1002/jcp.30145 33151563

[87] Yang T Cao C Yang J Liu T Lei XG Zhang Z miR-200a-5p regulates myocardial necroptosis induced by Se deficiency via targeting RNF11. *Redox Biol.* (2018) 15:159–69. 10.1016/j.redox.2017.11.025 29248830 PMC5975215

[88] Jiao L He Z Wang S Sun C Xu S. miR-130-CYLD axis is involved in the necroptosis and inflammation induced by selenium deficiency in pig cerebellum. *Biol Trace Elem Res.* (2021) 199:4604–13. 10.1007/s12011-021-02612-6 34331175

[89] Chen H Li P Shen Z Wang J Diao L. Protective effects of selenium yeast against cadmium-induced necroptosis through miR-26a-5p/PTEN/PI3K/AKT signaling pathway in chicken kidney. *Ecotoxicol Environ Saf.* (2021) 220:112387. 10.1016/j.ecoenv.2021.112387 34111659

[90] Zhang J Hao X Xu S. Selenium prevents lead-induced necroptosis by restoring antioxidant functions and blocking MAPK/NF-kappaB pathway in chicken lymphocytes. *Biol Trace Elem Res.* (2020) 198:644–53. 10.1007/s12011-020-02094-y 32279190

[91] Wang Y Li X Yao Y Zhao X Shi X Cai Y. Selenium deficiency induces apoptosis and necroptosis through ROS/MAPK signal in human uterine smooth muscle cells. *Biol Trace Elem Res.* (2021) 200:3147–3158. 10.1007/s12011-021-02910-z 34480665

[92] Zhang Y Yu D Zhang J Bao J Tang C Zhang Z. The role of necroptosis and apoptosis through the oxidative stress pathway in the liver of selenium-deficient swine. *Metallomics.* (2020) 12:607–16. 10.1039/c9mt00295b 32176230

[93] Sonkusre P Cameotra SS. Biogenic selenium nanoparticles induce ROS-mediated necroptosis in PC-3 cancer cells through TNF activation. *J Nanobiotechnol.* (2017) 15:43. 10.1186/s12951-017-0276-3 28592284 PMC5463494

[94] Sonkusre P. Specificity of biogenic selenium nanoparticles for prostate cancer therapy with reduced risk of toxicity: an in vitro and in vivo study. *Front Oncol.* (2019) 9:1541. 10.3389/fonc.2019.01541 32010628 PMC6978793

[95] Conrad M Pratt DA. The chemical basis of ferroptosis. *Nat Chem Biol.* (2019) 15:1137–47. 10.1038/s41589-019-0408-1 31740834

[96] Yao Y Chen Z Zhang H Chen C Zeng M Yunis J Selenium-GPX4 axis protects follicular helper T cells from ferroptosis. *Nat Immunol.* (2021) 22:1127–39. 10.1038/s41590-021-00996-0 34413521

[97] Tuo QZ Masaldan S Southon A Mawal C Ayton S Bush AI Characterization of selenium compounds for anti-ferroptotic activity in neuronal cells and after cerebral ischemia-reperfusion injury. *Neurotherapeutics.* (2021) 18:2682–91. 10.1007/s13311-021-01111-9 34498224 PMC8804037

[98] Alim I Caulfield JT Chen Y Swarup V Geschwind DH Ivanova E Selenium drives a transcriptional adaptive program to block ferroptosis and treat stroke. *Cell.* (2019) 177:1262–79.e25. 10.1016/j.cell.2019.03.032 31056284

[99] Liu L Wang M Gong N Tian P Deng H. Se improves GPX4 expression and SOD activity to alleviate heat-stress-induced ferroptosis-like death in goat mammary epithelial cells. *Anim Cells Syst (Seoul).* (2021) 25:283–95. 10.1080/19768354.2021.1988704 34745435 PMC8567913

[100] Day K Seale LA Graham RM Cardoso BR. Selenotranscriptome network in non-alcoholic fatty liver disease. *Front Nutr.* (2021) 8:744825. 10.3389/fnut.2021.744825 34869521 PMC8635790

[101] Vande Voorde J Ackermann T Pfetzer N Sumpton D Mackay G Kalna G Improving the metabolic fidelity of cancer models with a physiological cell culture medium. *Sci Adv.* (2019) 5:eaau7314. 10.1126/sciadv.aau7314 30613774 PMC6314821

[102] Belavgeni A Bornstein SR von Massenhausen A Tonnus W Stumpf J Meyer C Exquisite sensitivity of adrenocortical carcinomas to induction of ferroptosis. *Proc Natl Acad Sci U.S.A.* (2019) 116:22269–74. 10.1073/pnas.1912700116 31611400 PMC6825277

[103] Fan R Sui J Dong X Jing B Gao Z. Wedelolactone alleviates acute pancreatitis and associated lung injury via GPX4 mediated suppression of pyroptosis and ferroptosis. *Free Radic Biol Med.* (2021) 173:29–40. 10.1016/j.freeradbiomed.2021.07.009 34246777

[104] Wang S Liu W Wang J Bai X. Curculigoside inhibits ferroptosis in ulcerative colitis through the induction of GPX4. *Life Sci.* (2020) 259:118356. 10.1016/j.lfs.2020.118356 32861798

[105] Ingold I Berndt C Schmitt S Doll S Poschmann G Buday K Selenium utilization by GPX4 is required to prevent hydroperoxide-induced ferroptosis. *Cell.* (2018) 172:409–22.e21. 10.1016/j.cell.2017.11.048 29290465

[106] Ingold I Aichler M Yefremova E Roveri A Buday K Doll S Expression of a catalytically inactive mutant form of glutathione peroxidase 4 (Gpx4) confers a dominant-negative effect in male fertility. *J Biol Chem.* (2015) 290:14668–78. 10.1074/jbc.M115.656363 25922076 PMC4505533

[107] Liu H Forouhar F Seibt T Saneto R Wigby K Friedman J Characterization of a patient-derived variant of GPX4 for precision therapy. *Nat Chem Biol.* (2022) 18:91–100. 10.1038/s41589-021-00915-2 34931062 PMC8712418

[108] Lee N Carlisle AE Peppers A Park SJ Doshi MB Spears ME xCT-driven expression of GPX4 determines sensitivity of breast cancer cells to ferroptosis inducers. *Antioxidants (Basel).* (2021) 10:317. 10.3390/antiox10020317 33672555 PMC7923775

[109] Carlisle AE Lee N Matthew-Onabanjo AN Spears ME Park SJ Youkana D Selenium detoxification is required for cancer-cell survival. *Nat Metab.* (2020) 2:603–11. 10.1038/s42255-020-0224-7 32694795 PMC7455022

[110] Kitabayashi N Nakao S Mita Y Arisawa K Hoshi T Toyama T Role of selenoprotein P expression in the function of pancreatic beta cells: prevention of ferroptosis-like cell death and stress-induced nascent granule degradation. *Free Radic Biol Med.* (2022) 183:89–103. 10.1016/j.freeradbiomed.2022.03.009 35318102

[111] Rong Y Gao J Kuang T Chen J Li JA Huang Y DIAPH3 promotes pancreatic cancer progression by activating selenoprotein TrxR1-mediated antioxidant effects. *J Cell Mol Med.* (2021) 25:2163–75. 10.1111/jcmm.16196 33345387 PMC7882936

[112] Subburayan K Thayyullathil F Pallichankandy S Cheratta AR Galadari S. Superoxide-mediated ferroptosis in human cancer cells induced by sodium selenite. *Transl Oncol.* (2020) 13:100843. 10.1016/j.tranon.2020.100843 32805675 PMC7453065

[113] Fang X Ardehali H Min J Wang F. The molecular and metabolic landscape of iron and ferroptosis in cardiovascular disease. *Nat Rev Cardiol.* (2022). 10.1038/s41569-022-00735-4 [Epub ahead of print]. 35788564 PMC9252571

[114] Andrews NC. Iron metabolism: iron deficiency and iron overload. *Annu Rev Genomics Hum Genet.* (2000) 1:75–98. 10.1146/annurev.genom.1.1.75 11701625

[115] Nemeth E Ganz T. The role of hepcidin in iron metabolism. *Acta Haematol.* (2009) 122:78–86. 10.1159/000243791 19907144 PMC2855274

[116] Munoz M Garcia-Erce JA Remacha AF. Disorders of iron metabolism. Part II: iron deficiency and iron overload. *J Clin Pathol.* (2011) 64:287–96. 10.1136/jcp.2010.086991 21177268

[117] Conrad M Kagan VE Bayir H Pagnussat GC Head B Traber MG Regulation of lipid peroxidation and ferroptosis in diverse species. *Genes Dev.* (2018) 32:602–19. 10.1101/gad.314674.118 29802123 PMC6004068

[118] Stoyanovsky DA Tyurina YY Shrivastava I Bahar I Tyurin VA Protchenko O Iron catalysis of lipid peroxidation in ferroptosis: regulated enzymatic or random free radical reaction? *Free Radic Biol Med.* (2019) 133:153–61. 10.1016/j.freeradbiomed.2018.09.008 30217775 PMC6555767

[119] Ying JF Lu ZB Fu LQ Tong Y Wang Z Li WF The role of iron homeostasis and iron-mediated ROS in cancer. *Am J Cancer Res.* (2021) 11:1895–912. 34094660 PMC8167679

[120] Ravingerova T Kindernay L Bartekova M Ferko M Adameova A Zohdi V The molecular mechanisms of iron metabolism and its role in cardiac dysfunction and cardioprotection. *Int J Mol Sci.* (2020) 21:7889. 10.3390/ijms21217889 33114290 PMC7660609

[121] Kagan VE Mao G Qu F Angeli JP Doll S Croix CS Oxidized arachidonic and adrenic PEs navigate cells to ferroptosis. *Nat Chem Biol.* (2017) 13:81–90. 10.1038/nchembio.2238 27842066 PMC5506843

[122] Li Y Maher P Schubert D. A role for 12-lipoxygenase in nerve cell death caused by glutathione depletion. *Neuron.* (1997) 19:453–63. 10.1016/s0896-627380953-8 9292733

[123] Seiler A Schneider M Forster H Roth S Wirth EK Culmsee C Glutathione peroxidase 4 senses and translates oxidative stress into 12/15-lipoxygenase dependent- and AIF-mediated cell death. *Cell Metab.* (2008) 8:237–48. 10.1016/j.cmet.2008.07.005 18762024

[124] Zou Y Li H Graham ET Deik AA Eaton JK Wang W Cytochrome P450 oxidoreductase contributes to phospholipid peroxidation in ferroptosis. *Nat Chem Biol.* (2020) 16:302–9. 10.1038/s41589-020-0472-6 32080622 PMC7353921

[125] Lane DJ Merlot AM Huang ML Bae DH Jansson PJ Sahni S Cellular iron uptake, trafficking and metabolism: key molecules and mechanisms and their roles in disease. *Biochim Biophys Acta.* (2015) 1853:1130–44. 10.1016/j.bbamcr.2015.01.021 25661197

[126] Ohgami RS Campagna DR Greer EL Antiochos B McDonald A Chen J Identification of a ferrireductase required for efficient transferrin-dependent iron uptake in erythroid cells. *Nat Genet.* (2005) 37:1264–9. 10.1038/ng1658 16227996 PMC2156108

[127] Hider RC Kong XL. Glutathione: a key component of the cytoplasmic labile iron pool. *Biometals.* (2011) 24:1179–87. 10.1007/s10534-011-9476-8 21769609

[128] Leidgens S Bullough KZ Shi H Li F Shakoury-Elizeh M Yabe T Each member of the poly-r(C)-binding protein 1 (PCBP) family exhibits iron chaperone activity toward ferritin. *J Biol Chem.* (2013) 288:17791–802. 10.1074/jbc.M113.460253 23640898 PMC3682578

[129] Shi H Bencze KZ Stemmler TL Philpott CC. A cytosolic iron chaperone that delivers iron to ferritin. *Science.* (2008) 320:1207–10. 10.1126/science.1157643 18511687 PMC2505357

[130] Chen X Yu C Kang R Tang D. Iron metabolism in ferroptosis. *Front Cell Dev Biol.* (2020) 8:590226. 10.3389/fcell.2020.590226 33117818 PMC7575751

[131] Arosio P Levi S. Ferritin, iron homeostasis, and oxidative damage. *Free Radic Biol Med.* (2002) 33:457–63. 10.1016/s0891-584900842-0 12160928

[132] Arosio P Elia L Poli M. Ferritin, cellular iron storage and regulation. *IUBMB Life.* (2017) 69:414–22. 10.1002/iub.1621 28349628

[133] Park E Chung SW. ROS-mediated autophagy increases intracellular iron levels and ferroptosis by ferritin and transferrin receptor regulation. *Cell Death Dis.* (2019) 10:822. 10.1038/s41419-019-2064-5 31659150 PMC6817894

[134] Fang X Cai Z Wang H Han D Cheng Q Zhang P Loss of cardiac ferritin H facilitates cardiomyopathy via Slc7a11-mediated ferroptosis. *Circ Res.* (2020) 127:486–501. 10.1161/CIRCRESAHA.120.316509 32349646

[135] Wang YQ Chang SY Wu Q Gou YJ Jia L Cui YM The protective role of mitochondrial ferritin on erastin-induced ferroptosis. *Front Aging Neurosci.* (2016) 8:308. 10.3389/fnagi.2016.00308 28066232 PMC5167726

[136] Wang P Cui Y Ren Q Yan B Zhao Y Yu P Mitochondrial ferritin attenuates cerebral ischaemia/reperfusion injury by inhibiting ferroptosis. *Cell Death Dis.* (2021) 12:447. 10.1038/s41419-021-03725-5 33953171 PMC8099895

[137] Wang X Ma H Sun J Zheng T Zhao P Li H Mitochondrial ferritin deficiency promotes osteoblastic ferroptosis via mitophagy in type 2 diabetic osteoporosis. *Biol Trace Elem Res.* (2022) 200:298–307. 10.1007/s12011-021-02627-z 33594527

[138] Hou W Xie Y Song X Sun X Lotze MT Zeh HJ III Autophagy promotes ferroptosis by degradation of ferritin. *Autophagy.* (2016) 12:1425–8. 10.1080/15548627.2016.1187366 27245739 PMC4968231

[139] Fuhrmann DC Mondorf A Beifuss J Jung M Brune B. Hypoxia inhibits ferritinophagy, increases mitochondrial ferritin, and protects from ferroptosis. *Redox Biol.* (2020) 36:101670. 10.1016/j.redox.2020.101670 32810738 PMC7452134

[140] Gao M Monian P Pan Q Zhang W Xiang J Jiang X. Ferroptosis is an autophagic cell death process. *Cell Res.* (2016) 26:1021–32. 10.1038/cr.2016.95 27514700 PMC5034113

[141] Chen GQ Benthani FA Wu J Liang D Bian ZX Jiang X. Artemisinin compounds sensitize cancer cells to ferroptosis by regulating iron homeostasis. *Cell Death Differ.* (2020) 27:242–54. 10.1038/s41418-019-0352-3 31114026 PMC7205875

[142] Li N Wang W Zhou H Wu Q Duan M Liu C Ferritinophagy-mediated ferroptosis is involved in sepsis-induced cardiac injury. *Free Radic Biol Med.* (2020) 160:303–18. 10.1016/j.freeradbiomed.2020.08.009 32846217

[143] Sun X Ou Z Chen R Niu X Chen D Kang R Activation of the p62-Keap1-NRF2 pathway protects against ferroptosis in hepatocellular carcinoma cells. *Hepatology.* (2016) 63:173–84. 10.1002/hep.28251 26403645 PMC4688087

[144] Wang X Chen X Zhou W Men H Bao T Sun Y Ferroptosis is essential for diabetic cardiomyopathy and is prevented by sulforaphane via AMPK/NRF2 pathways. *Acta Pharm Sin B.* (2022) 12:708–22. 10.1016/j.apsb.2021.10.005 35256941 PMC8897044

[145] Li X Si W Li Z Tian Y Liu X Ye S miR335 promotes ferroptosis by targeting ferritin heavy chain 1 in in vivo and in vitro models of Parkinson’s disease. *Int J Mol Med.* (2021) 47:61. 10.3892/ijmm.2021.4894 33649797 PMC7910012

[146] Brown CW Amante JJ Chhoy P Elaimy AL Liu H Zhu LJ Prominin2 drives ferroptosis resistance by stimulating iron export. *Dev Cell.* (2019) 51:575–86.e4. 10.1016/j.devcel.2019.10.007 31735663 PMC8316835

[147] Wang CY Knutson MD. Hepatocyte divalent metal-ion transporter-1 is dispensable for hepatic iron accumulation and non-transferrin-bound iron uptake in mice. *Hepatology.* (2013) 58:788–98. 10.1002/hep.26401 23508576 PMC4572840

[148] Yu Y Jiang L Wang H Shen Z Cheng Q Zhang P Hepatic transferrin plays a role in systemic iron homeostasis and liver ferroptosis. *Blood.* (2020) 136:726–39. 10.1182/blood.2019002907 32374849 PMC7414596

[149] Hong X Roh W Sullivan RJ Wong KHK Wittner BS Guo H The lipogenic regulator SREBP2 induces transferrin in circulating melanoma cells and suppresses ferroptosis. *Cancer Discov.* (2021) 11:678–95. 10.1158/2159-8290.CD-19-1500 33203734 PMC7933049

[150] Gao M Monian P Quadri N Ramasamy R Jiang X. Glutaminolysis and transferrin regulate ferroptosis. *Mol Cell.* (2015) 59:298–308. 10.1016/j.molcel.2015.06.011 26166707 PMC4506736

[151] Song J Liu T Yin Y Zhao W Lin Z Yin Y The deubiquitinase OTUD1 enhances iron transport and potentiates host antitumor immunity. *EMBO Rep.* (2021) 22:e51162. 10.15252/embr.202051162 33393230 PMC7857436

[152] Tang LJ Zhou YJ Xiong XM Li NS Zhang JJ Luo XJ Ubiquitin-specific protease 7 promotes ferroptosis via activation of the p53/TfR1 pathway in the rat hearts after ischemia/reperfusion. *Free Radic Biol Med.* (2021) 162:339–52. 10.1016/j.freeradbiomed.2020.10.307 33157209

[153] Wu Y Jiao H Yue Y He K Jin Y Zhang J Ubiquitin ligase E3 HUWE1/MULE targets transferrin receptor for degradation and suppresses ferroptosis in acute liver injury. *Cell Death Differ.* (2022) 29:1705–1718. 10.1038/s41418-022-00957-6 35260822 PMC9433446

[154] Bao WD Pang P Zhou XT Hu F Xiong W Chen K Loss of ferroportin induces memory impairment by promoting ferroptosis in Alzheimer’s disease. *Cell Death Differ.* (2021) 28:1548–62. 10.1038/s41418-020-00685-9 33398092 PMC8166828

[155] Bao WD Zhou XT Zhou LT Wang F Yin X Lu Y Targeting miR-124/Ferroportin signaling ameliorated neuronal cell death through inhibiting apoptosis and ferroptosis in aged intracerebral hemorrhage murine model. *Aging Cell.* (2020) 19:e13235. 10.1111/acel.13235 33068460 PMC7681046

[156] Basuli D Tesfay L Deng Z Paul B Yamamoto Y Ning G Iron addiction: a novel therapeutic target in ovarian cancer. *Oncogene.* (2017) 36:4089–99. 10.1038/onc.2017.11 28319068 PMC5540148

[157] Yao F Cui X Zhang Y Bei Z Wang H Zhao D Iron regulatory protein 1 promotes ferroptosis by sustaining cellular iron homeostasis in melanoma. *Oncol Lett.* (2021) 22:657. 10.3892/ol.2021.12918 34386079 PMC8299017

[158] Wu J Minikes AM Gao M Bian H Li Y Stockwell BR Intercellular interaction dictates cancer cell ferroptosis via NF2-YAP signalling. *Nature.* (2019) 572:402–6. 10.1038/s41586-019-1426-6 31341276 PMC6697195

[159] Zhang X Yu K Ma L Qian Z Tian X Miao Y Endogenous glutamate determines ferroptosis sensitivity via ADCY10-dependent YAP suppression in lung adenocarcinoma. *Theranostics.* (2021) 11:5650–74. 10.7150/thno.55482 33897873 PMC8058707

[160] Wang Y Qiu S Wang H Cui J Tian X Miao Y Transcriptional repression of ferritin light chain increases ferroptosis sensitivity in lung adenocarcinoma. *Front Cell Dev Biol.* (2021) 9:719187. 10.3389/fcell.2021.719187 34765600 PMC8576304

[161] Chen PH Wu J Ding CC Lin CC Pan S Bossa N Kinome screen of ferroptosis reveals a novel role of ATM in regulating iron metabolism. *Cell Death Differ.* (2020) 27:1008–22. 10.1038/s41418-019-0393-7 31320750 PMC7206124

[162] Ciofi-Baffoni S Nasta V Banci L. Protein networks in the maturation of human iron-sulfur proteins. *Metallomics.* (2018) 10:49–72. 10.1039/c7mt00269f 29219157

[163] Lee J You JH Shin D Roh JL. Inhibition of glutaredoxin 5 predisposes cisplatin-resistant head and neck cancer cells to ferroptosis. *Theranostics.* (2020) 10:7775–86. 10.7150/thno.46903 32685019 PMC7359084

[164] Dai MC Zhong ZH Sun YH Sun QF Wang YT Yang GY Curcumin protects against iron induced neurotoxicity in primary cortical neurons by attenuating necroptosis. *Neurosci Lett.* (2013) 536:41–6. 10.1016/j.neulet.2013.01.007 23328441

[165] Daruich A Le Rouzic Q Jonet L Naud MC Kowalczuk L Pournaras JA Iron is neurotoxic in retinal detachment and transferrin confers neuroprotection. *Sci Adv.* (2019) 5:eaau9940. 10.1126/sciadv.aau9940 30662950 PMC6326753

[166] Tian Q Qin B Gu Y Zhou L Chen S Zhang S ROS-mediated necroptosis is involved in iron overload-induced osteoblastic cell death. *Oxid Med Cell Longev.* (2020) 2020:1295382. 10.1155/2020/1295382 33123307 PMC7586162

[167] Figueiredo RT Fernandez PL Mourao-Sa DS Porto BN Dutra FF Alves LS Characterization of heme as activator of toll-like receptor 4. *J Biol Chem.* (2007) 282:20221–9. 10.1074/jbc.M610737200 17502383

[168] Fortes GB Alves LS de Oliveira R Dutra FF Rodrigues D Fernandez PL Heme induces programmed necrosis on macrophages through autocrine TNF and ROS production. *Blood.* (2012) 119:2368–75. 10.1182/blood-2011-08-375303 22262768 PMC3358230

[169] Zhou B Zhang JY Liu XS Chen HZ Ai YL Cheng K Tom20 senses iron-activated ROS signaling to promote melanoma cell pyroptosis. *Cell Res.* (2018) 28:1171–85. 10.1038/s41422-018-0090-y 30287942 PMC6274649

[170] Hwang C Ross V Mahadevan U. Micronutrient deficiencies in inflammatory bowel disease: from A to zinc. *Inflamm Bowel Dis.* (2012) 18:1961–81. 10.1002/ibd.22906 22488830

[171] Andreini C Banci L Bertini I Rosato A. Counting the zinc-proteins encoded in the human genome. *J Proteome Res.* (2006) 5:196–201. 10.1021/pr050361j 16396512

[172] Wessels I Fischer HJ Rink L. Dietary and physiological effects of zinc on the immune system. *Annu Rev Nutr.* (2021) 41:133–75. 10.1146/annurev-nutr-122019-120635 34255547

[173] Sanna A Firinu D Zavattari P Valera P. Zinc status and autoimmunity: a systematic review and meta-analysis. *Nutrients.* (2018) 10:68. 10.3390/nu10010068 29324654 PMC5793296

[174] Fukada T Yamasaki S Nishida K Murakami M Hirano T. Zinc homeostasis and signaling in health and diseases: zinc signaling. *J Biol Inorg Chem.* (2011) 16:1123–34. 10.1007/s00775-011-0797-4 21660546 PMC3176402

[175] Tokuyama A Kanda E Itano S Kondo M Wada Y Kadoya H Effect of zinc deficiency on chronic kidney disease progression and effect modification by hypoalbuminemia. *PLoS One.* (2021) 16:e0251554. 10.1371/journal.pone.0251554 33974681 PMC8112700

[176] Palmer LD Jordan AT Maloney KN Farrow MA Gutierrez DB Gant-Branum R Zinc intoxication induces ferroptosis in A549 human lung cells. *Metallomics.* (2019) 11:982–93. 10.1039/c8mt00360b 30968088 PMC6531343

[177] Chen PH Wu J Xu Y Ding CC Mestre AA Lin CC Zinc transporter ZIP7 is a novel determinant of ferroptosis. *Cell Death Dis.* (2021) 12:198. 10.1038/s41419-021-03482-5 33608508 PMC7895949

[178] Zhang C Liu Z Zhang Y Ma L Song E Song Y. “Iron free” zinc oxide nanoparticles with ion-leaking properties disrupt intracellular ROS and iron homeostasis to induce ferroptosis. *Cell Death Dis.* (2020) 11:183. 10.1038/s41419-020-2384-5 32170066 PMC7070056

[179] Meng X Deng J Liu F Guo T Liu M Dai P Triggered all-active metal organic framework: ferroptosis machinery contributes to the apoptotic photodynamic antitumor therapy. *Nano Lett.* (2019) 19:7866–76. 10.1021/acs.nanolett.9b02904 31594301

[180] Qin X Zhang J Wang B Xu G Yang X Zou Z Ferritinophagy is involved in the zinc oxide nanoparticles-induced ferroptosis of vascular endothelial cells. *Autophagy.* (2021) 17:4266–85. 10.1080/15548627.2021.1911016 33843441 PMC8726675

[181] Qin X Tang Q Jiang X Zhang J Wang B Liu X Zinc oxide nanoparticles induce ferroptotic neuronal cell death in vitro and in vivo. *Int J Nanomedicine.* (2020) 15:5299–315. 10.2147/IJN.S250367 32884256 PMC7436556

[182] Li Y Li J Li Z Wei M Zhao H Miyagishi M Homeostasis imbalance of YY2 and YY1 promotes tumor growth by manipulating ferroptosis. *Adv Sci (Weinh).* (2022) 9:e2104836. 10.1002/advs.202104836 35246964 PMC9069185

[183] Xiao FJ Zhang D Wu Y Jia QH Zhang L Li YX miRNA-17-92 protects endothelial cells from erastin-induced ferroptosis through targeting the A20-ACSL4 axis. *Biochem Biophys Res Commun.* (2019) 515:448–54. 10.1016/j.bbrc.2019.05.147 31160087

[184] Hsieh H Vignesh KS Deepe GS Jr Choubey D Shertzer HG Genter MB. Mechanistic studies of the toxicity of zinc gluconate in the olfactory neuronal cell line Odora. *Toxicol Vitro.* (2016) 35:24–30. 10.1016/j.tiv.2016.05.003 27179668 PMC5097460

[185] Chen YY Lee YH Wang BJ Chen RJ Wang YJ. Skin damage induced by zinc oxide nanoparticles combined with UVB is mediated by activating cell pyroptosis via the NLRP3 inflammasome-autophagy-exosomal pathway. *Part Fibre Toxicol.* (2022) 19:2. 10.1186/s12989-021-00443-w 34983566 PMC8729117

[186] Le X Mu J Peng W Tang J Xiang Q Tian S DNA methylation downregulated ZDHHC1 suppresses tumor growth by altering cellular metabolism and inducing oxidative/ER stress-mediated apoptosis and pyroptosis. *Theranostics.* (2020) 10:9495–511. 10.7150/thno.45631 32863941 PMC7449911

[187] He S Ma C Zhang L Bai J Wang X Zheng X GLI1-mediated pulmonary artery smooth muscle cell pyroptosis contributes to hypoxia-induced pulmonary hypertension. *Am J Physiol Lung Cell Mol Physiol.* (2020) 318:L472–82. 10.1152/ajplung.00405.2019 31868509

[188] Liu JJ Li Y Yang MS Chen R Cen CQ. SP1-induced ZFAS1 aggravates sepsis-induced cardiac dysfunction via miR-590-3p/NLRP3-mediated autophagy and pyroptosis. *Arch Biochem Biophys.* (2020) 695:108611. 10.1016/j.abb.2020.108611 33002446

[189] Fauster A Rebsamen M Willmann KL Cesar-Razquin A Girardi E Bigenzahn JW Systematic genetic mapping of necroptosis identifies SLC39A7 as modulator of death receptor trafficking. *Cell Death Differ.* (2019) 26:1138–55. 10.1038/s41418-018-0192-6 30237509 PMC6748104

[190] Zhong Y Zhang ZH Wang JY Xing Y Ri MH Jin HL Zinc finger protein 91 mediates necroptosis by initiating RIPK1-RIPK3-MLKL signal transduction in response to TNF receptor 1 ligation. *Toxicol Lett.* (2022) 356:75–88. 10.1016/j.toxlet.2021.12.015 34942311

[191] Yang C Li J Yu L Zhang Z Xu F Jiang L Regulation of RIP3 by the transcription factor Sp1 and the epigenetic regulator UHRF1 modulates cancer cell necroptosis. *Cell Death Dis.* (2017) 8:e3084. 10.1038/cddis.2017.483 28981102 PMC5682651

[192] Souffriau J Timmermans S Vanderhaeghen T Wallaeys C Van Looveren K Aelbrecht L Zinc inhibits lethal inflammatory shock by preventing microbe-induced interferon signature in intestinal epithelium. *EMBO Mol Med.* (2020) 12:e11917. 10.15252/emmm.201911917 32914580 PMC7539219

[193] Ge MH Tian H Mao L Li DY Lin JQ Hu HS Zinc attenuates ferroptosis and promotes functional recovery in contusion spinal cord injury by activating Nrf2/GPX4 defense pathway. *CNS Neurosci Ther.* (2021) 27:1023–40. 10.1111/cns.13657 33951302 PMC8339532

[194] Polykratis A Martens A Eren RO Shirasaki Y Yamagishi M Yamaguchi Y A20 prevents inflammasome-dependent arthritis by inhibiting macrophage necroptosis through its ZnF7 ubiquitin-binding domain. *Nat Cell Biol.* (2019) 21:731–42. 10.1038/s41556-019-0324-3 31086261

[195] Zhang X Zheng Q Yue X Yuan Z Ling J Yuan Y ZNF498 promotes hepatocellular carcinogenesis by suppressing p53-mediated apoptosis and ferroptosis via the attenuation of p53 Ser46 phosphorylation. *J Exp Clin Cancer Res.* (2022) 41:79. 10.1186/s13046-022-02288-3 35227287 PMC8883630

[196] Zhao Z Hu X Wang J Wang J Hou Y Chen S. Zinc finger E-box binding homeobox 2 (ZEB2)-induced astrogliosis protected neuron from pyroptosis in cerebral ischemia and reperfusion injury. *Bioengineered.* (2021) 12:12917–30. 10.1080/21655979.2021.2012551 34852714 PMC8809936

[197] Jiang Q. Natural forms of vitamin E: metabolism, antioxidant, and anti-inflammatory activities and their role in disease prevention and therapy. *Free Radic Biol Med.* (2014) 72:76–90. 10.1016/j.freeradbiomed.2014.03.035 24704972 PMC4120831

[198] Traber MG Atkinson J. Vitamin E, antioxidant and nothing more. *Free Radic Biol Med.* (2007) 43:4–15. 10.1016/j.freeradbiomed.2007.03.024 17561088 PMC2040110

[199] Kajarabille N Latunde-Dada GO. Programmed cell-death by ferroptosis: antioxidants as mitigators. *Int J Mol Sci.* (2019) 20:4968. 10.3390/ijms20194968 31597407 PMC6801403

[200] Wortmann M Schneider M Pircher J Hellfritsch J Aichler M Vegi N Combined deficiency in glutathione peroxidase 4 and vitamin E causes multiorgan thrombus formation and early death in mice. *Circ Res.* (2013) 113:408–17. 10.1161/CIRCRESAHA.113.279984 23770613

[201] Carlson BA Tobe R Yefremova E Tsuji PA Hoffmann VJ Schweizer U Glutathione peroxidase 4 and vitamin E cooperatively prevent hepatocellular degeneration. *Redox Biol.* (2016) 9:22–31. 10.1016/j.redox.2016.05.003 27262435 PMC4900515

[202] Matsushita M Freigang S Schneider C Conrad M Bornkamm GW Kopf M. T cell lipid peroxidation induces ferroptosis and prevents immunity to infection. *J Exp Med.* (2015) 212:555–68. 10.1084/jem.20140857 25824823 PMC4387287

[203] Altamura S Vegi NM Hoppe PS Schroeder T Aichler M Walch A Glutathione peroxidase 4 and vitamin E control reticulocyte maturation, stress erythropoiesis and iron homeostasis. *Haematologica.* (2020) 105:937–50. 10.3324/haematol.2018.212977 31248967 PMC7109755

[204] Chen L Hambright WS Na R Ran Q. Ablation of the ferroptosis inhibitor glutathione peroxidase 4 in neurons results in rapid motor neuron degeneration and paralysis. *J Biol Chem.* (2015) 290:28097–106. 10.1074/jbc.M115.680090 26400084 PMC4653669

[205] Sakai O Yasuzawa T Sumikawa Y Ueta T Imai H Sawabe A Role of GPx4 in human vascular endothelial cells, and the compensatory activity of brown rice on GPx4 ablation condition. *Pathophysiology.* (2017) 24:9–15. 10.1016/j.pathophys.2016.11.002 27964880

[206] Hu Q Zhang Y Lou H Ou Z Liu J Duan W GPX4 and vitamin E cooperatively protect hematopoietic stem and progenitor cells from lipid peroxidation and ferroptosis. *Cell Death Dis.* (2021) 12:706. 10.1038/s41419-021-04008-9 34267193 PMC8282880

[207] Hambright WS Fonseca RS Chen L Na R Ran Q. Ablation of ferroptosis regulator glutathione peroxidase 4 in forebrain neurons promotes cognitive impairment and neurodegeneration. *Redox Biol.* (2017) 12:8–17. 10.1016/j.redox.2017.01.021 28212525 PMC5312549

[208] Matsuo T dachi-Tominari KA Sano O Kamei T Nogami M Ogi K Involvement of ferroptosis in human motor neuron cell death. *Biochem Biophys Res Commun.* (2021) 566:24–9. 10.1016/j.bbrc.2021.05.095 34111668

[209] Azuma K Koumura T Iwamoto R Matsuoka M Terauchi R Yasuda S Mitochondrial glutathione peroxidase 4 is indispensable for photoreceptor development and survival in mice. *J Biol Chem.* (2022) 298:101824. 10.1016/j.jbc.2022.101824 35288190 PMC8980337

[210] Feng H Stockwell BR. Unsolved mysteries: how does lipid peroxidation cause ferroptosis? *PLoS Biol.* (2018) 16:e2006203. 10.1371/journal.pbio.2006203 29795546 PMC5991413

[211] Hinman A Holst CR Latham JC Bruegger JJ Ulas G McCusker KP Vitamin E hydroquinone is an endogenous regulator of ferroptosis via redox control of 15-lipoxygenase. *PLoS One.* (2018) 13:e0201369. 10.1371/journal.pone.0201369 30110365 PMC6093661

[212] Zhang X Wu S Guo C Guo K Hu Z Peng J Vitamin E exerts neuroprotective effects in pentylenetetrazole kindling epilepsy via suppression of ferroptosis. *Neurochem Res.* (2022) 47:739–47. 10.1007/s11064-021-03483-y 34779994

[213] Protchenko O Baratz E Jadhav S Li F Shakoury-Elizeh M Gavrilova O Iron chaperone poly rC binding protein 1 protects mouse liver from lipid peroxidation and steatosis. *Hepatology.* (2021) 73:1176–93. 32438524 10.1002/hep.31328 PMC8364740

[214] Jadhav S Protchenko O Li F Baratz E Shakoury-Elizeh M Maschek A Mitochondrial dysfunction in mouse livers depleted of iron chaperone PCBP1. *Free Radic Biol Med.* (2021) 175:18–27. 10.1016/j.freeradbiomed.2021.08.232 34455040 PMC9137418

[215] Kang R Zeng L Zhu S Xie Y Liu J Wen Q Lipid peroxidation drives gasdermin D-mediated pyroptosis in lethal polymicrobial sepsis. *Cell Host Microbe.* (2018) 24:97–108e4. 10.1016/j.chom.2018.05.009 29937272 PMC6043361

[216] Jian L Xue Y Gao Y Wang B Qu Y Li S Vitamin E can ameliorate oxidative damage of ovine hepatocytes in vitro by regulating genes expression associated with apoptosis and pyroptosis, but not ferroptosis. *Molecules.* (2021) 26:4520. 10.3390/molecules26154520 34361674 PMC8348559

[217] Schenk B Fulda S. Reactive oxygen species regulate Smac mimetic/TNFalpha-induced necroptotic signaling and cell death. *Oncogene.* (2015) 34:5796–806. 10.1038/onc.2015.35 25867066

[218] Basit F van Oppen LM Schockel L Bossenbroek HM van Emst-de Vries SE Hermeling JC Mitochondrial complex I inhibition triggers a mitophagy-dependent ROS increase leading to necroptosis and ferroptosis in melanoma cells. *Cell Death Dis.* (2017) 8:e2716. 10.1038/cddis.2017.133 28358377 PMC5386536

[219] Basit S. Vitamin D in health and disease: a literature review. *Br J Biomed Sci.* (2013) 70:161–72. 10.1080/09674845.2013.11669951 24400428

[220] Zittermann A Trummer C Theiler-Schwetz V Lerchbaum E Marz W Pilz S. Vitamin D and cardiovascular disease: an updated narrative review. *Int J Mol Sci.* (2021) 22:2896. 10.3390/ijms22062896 33809311 PMC7998446

[221] Pike JW Meyer MB. The vitamin D receptor: new paradigms for the regulation of gene expression by 1,25-dihydroxyvitamin D. *Endocrinol Metab Clin North Am.* (2010) 39:255–69, table of contents. 10.1016/j.ecl.2010.02.007 20511050 PMC2879406

[222] Cheng K Huang Y Wang C. 1,25(OH)2D3 inhibited ferroptosis in zebrafish liver cells (ZFL) by regulating Keap1-Nrf2-GPx4 and NF-kappaB-hepcidin axis. *Int J Mol Sci.* (2021) 22:11334. 10.3390/ijms222111334 34768761 PMC8583391

[223] Hu Z Zhang H Yi B Yang S Liu J Hu J VDR activation attenuate cisplatin induced AKI by inhibiting ferroptosis. *Cell Death Dis.* (2020) 11:73. 10.1038/s41419-020-2256-z 31996668 PMC6989512

[224] Shi Y Cui X Sun Y Zhao Q Liu T. Intestinal vitamin D receptor signaling ameliorates dextran sulfate sodium-induced colitis by suppressing necroptosis of intestinal epithelial cells. *FASEB J.* (2020) 34:13494–506. 10.1096/fj.202000143RRR 32779265

[225] Quarni W Lungchukiet P Tse A Wang P Sun Y Kasiappan R RIPK1 binds to vitamin D receptor and decreases vitamin D-induced growth suppression. *J Steroid Biochem Mol Biol.* (2017) 173:157–67. 10.1016/j.jsbmb.2017.01.024 28159673 PMC5538941

[226] Wu W Liu D Zhao Y Zhang T Ma J Wang D Cholecalciferol pretreatment ameliorates ischemia/reperfusion-induced acute kidney injury through inhibiting ROS production, NF-kappaB pathway and pyroptosis. *Acta Histochem.* (2022) 124:151875. 10.1016/j.acthis.2022.151875 35334282

[227] Jiang S Zhang H Li X Yi B Huang L Hu Z Vitamin D/VDR attenuate cisplatin-induced AKI by down-regulating NLRP3/Caspase-1/GSDMD pyroptosis pathway. *J Steroid Biochem Mol Biol.* (2021) 206:105789. 10.1016/j.jsbmb.2020.105789 33259938

[228] Zhang X Shang X Jin S Ma Z Wang H Ao N Vitamin D ameliorates high-fat-diet-induced hepatic injury via inhibiting pyroptosis and alters gut microbiota in rats. *Arch Biochem Biophys.* (2021) 705:108894. 10.1016/j.abb.2021.108894 33965368

[229] Huang Z Zhang Q Wang Y Chen R Wang Y Huang Z Inhibition of caspase-3-mediated GSDME-derived pyroptosis aids in noncancerous tissue protection of squamous cell carcinoma patients during cisplatin-based chemotherapy. *Am J Cancer Res.* (2020) 10:4287–307. 33415000 PMC7783734

[230] Wu M Lu L Guo K Lu J Chen H. Vitamin D protects against high glucose-induced pancreatic beta-cell dysfunction via AMPK-NLRP3 inflammasome pathway. *Mol Cell Endocrinol.* (2022) 547:111596. 10.1016/j.mce.2022.111596 35183675

[231] Pi Y Tian X Ma J Zhang H Huang X. Vitamin D alleviates hypoxia/reoxygenation-induced injury of human trophoblast HTR-8 cells by activating autophagy. *Placenta.* (2021) 111:10–8. 10.1016/j.placenta.2021.05.008 34126416

[232] Toth SZ Lorincz T Szarka A. Concentration does matter: the beneficial and potentially harmful effects of ascorbate in humans and plants. *Antioxid Redox Signal.* (2018) 29:1516–33. 10.1089/ars.2017.7125 28974112

[233] Szarka A Tomasskovics B Banhegyi G. The ascorbate-glutathione-alpha-tocopherol triad in abiotic stress response. *Int J Mol Sci.* (2012) 13:4458–83. 10.3390/ijms13044458 22605990 PMC3344226

[234] Shen J Griffiths PT Campbell SJ Utinger B Kalberer M Paulson SE. Ascorbate oxidation by iron, copper and reactive oxygen species: review, model development, and derivation of key rate constants. *Sci Rep.* (2021) 11:7417. 10.1038/s41598-021-86477-8 33795736 PMC8016884

[235] Wang X Xu S Zhang L Cheng X Yu H Bao J Vitamin C induces ferroptosis in anaplastic thyroid cancer cells by ferritinophagy activation. *Biochem Biophys Res Commun.* (2021) 551:46–53. 10.1016/j.bbrc.2021.02.126 33714759

[236] Rouleau L Antony AN Bisetto S Newberg A Doria C Levine M Synergistic effects of ascorbate and sorafenib in hepatocellular carcinoma: new insights into ascorbate cytotoxicity. *Free Radic Biol Med.* (2016) 95:308–22. 10.1016/j.freeradbiomed.2016.03.031 27036367 PMC4867251

[237] Lorenzato A Magri A Matafora V Audrito V Arcella P Lazzari L Vitamin C restricts the emergence of acquired resistance to egfr-targeted therapies in colorectal cancer. *Cancers (Basel).* (2020) 12:685. 10.3390/cancers12030685 32183295 PMC7140052

[238] Ferrada L Barahona MJ Salazar K Vandenabeele P Nualart F. Vitamin C controls neuronal necroptosis under oxidative stress. *Redox Biol.* (2020) 29:101408. 10.1016/j.redox.2019.101408 31926631 PMC6938857

[239] Li CJ Sun LY Pang CY. Synergistic protection of N-acetylcysteine and ascorbic acid 2-phosphate on human mesenchymal stem cells against mitoptosis, necroptosis and apoptosis. *Sci Rep.* (2015) 5:9819. 10.1038/srep09819 25909282 PMC4408980

[240] Tian W Wang Z Tang NN Li JT Liu Y Chu WF Ascorbic acid sensitizes colorectal carcinoma to the cytotoxicity of arsenic trioxide via promoting reactive oxygen species-dependent apoptosis and pyroptosis. *Front Pharmacol.* (2020) 11:123. 10.3389/fphar.2020.00123 32153415 PMC7047232

[241] Shan M Yu X Li Y Fu C Zhang C. Vitamin B6 alleviates lipopolysaccharide-induced myocardial injury by ferroptosis and apoptosis regulation. *Front Pharmacol.* (2021) 12:766820. 10.3389/fphar.2021.766820 35002705 PMC8740299

[242] Yang W Liu S Li Y Wang Y Deng Y Sun W Pyridoxine induces monocyte-macrophages death as specific treatment of acute myeloid leukemia. *Cancer Lett.* (2020) 492:96–105. 10.1016/j.canlet.2020.08.018 32860849

[243] Sotler R Poljsak B Dahmane R Jukic T Pavan Jukic D Rotim C Prooxidant activities of antioxidants and their impact on health. *Acta Clin Croat.* (2019) 58:726–36. 10.20471/acc.2019.58.04.20 32595258 PMC7314298

[244] Podmore ID Griffiths HR Herbert KE Mistry N Mistry P Lunec J. Vitamin C exhibits pro-oxidant properties. *Nature.* (1998) 392:559. 10.1038/33308 9560150

[245] Halliwell B. Vitamin C: antioxidant or pro-oxidant in vivo? *Free Radic Res.* (1996) 25:439–54. 10.3109/10715769609149066 8902542

[246] Chareonpong-Kawamoto N Yasumoto K. Selenium deficiency as a cause of overload of iron and unbalanced distribution of other minerals. *Biosci Biotechnol Biochem.* (1995) 59:302–6. 10.1271/bbb.59.302 7766029

[247] Ergul AB Turanoglu C Karakukcu C Karaman S Torun YA. Increased iron deficiency and iron deficiency anemia in children with zinc deficiency. *Eurasian J Med.* (2018) 50:34–7. 10.5152/eurasianjmed.2017.17237 29531489 PMC5843450

[248] Ma AG Chen XC Xu RX Zheng MC Wang Y Li JS. Comparison of serum levels of iron, zinc and copper in anaemic and non-anaemic pregnant women in China. *Asia Pac J Clin Nutr.* (2004) 13:348–52. 15563439

[249] Niles BJ Clegg MS Hanna LA Chou SS Momma TY Hong H Zinc deficiency-induced iron accumulation, a consequence of alterations in iron regulatory protein-binding activity, iron transporters, and iron storage proteins. *J Biol Chem.* (2008) 283:5168–77. 10.1074/jbc.M709043200 18073202

[250] Zheng D Kille P Feeney GP Cunningham P Handy RD Hogstrand C. Dynamic transcriptomic profiles of zebrafish gills in response to zinc supplementation. *BMC Genomics.* (2010) 11:553. 10.1186/1471-2164-11-553 20937081 PMC3091702

[251] Kondaiah P Yaduvanshi PS Sharp PA Pullakhandam R. Iron and zinc homeostasis and interactions: does enteric zinc excretion cross-talk with intestinal iron absorption? *Nutrients.* (2019) 11:1885. 10.3390/nu11081885 31412634 PMC6722515

[252] Lawen A Lane DJ. Mammalian iron homeostasis in health and disease: uptake, storage, transport, and molecular mechanisms of action. *Antioxid Redox Signal.* (2013) 18:2473–507. 10.1089/ars.2011.4271 23199217

[253] Lane DJ Richardson DR. The active role of vitamin C in mammalian iron metabolism: much more than just enhanced iron absorption! *Free Radic Biol Med.* (2014) 75:69–83. 10.1016/j.freeradbiomed.2014.07.007 25048971

[254] Toth I Bridges KR. Ascorbic acid enhances ferritin mRNA translation by an IRP/aconitase switch. *J Biol Chem.* (1995) 270:19540–4. 10.1074/jbc.270.33.19540 7642638

[255] Bridges KR. Ascorbic acid inhibits lysosomal autophagy of ferritin. *J Biol Chem.* (1987) 262:14773–8. 3667602

[256] Conrad M Angeli JP Vandenabeele P Stockwell BR. Regulated necrosis: disease relevance and therapeutic opportunities. *Nat Rev Drug Discov.* (2016) 15:348–66. 10.1038/nrd.2015.6 26775689 PMC6531857

[257] Reichert CO de Freitas FA Sampaio-Silva J Rokita-Rosa L Barros PL Levy D Ferroptosis mechanisms involved in neurodegenerative diseases. *Int J Mol Sci.* (2020) 21:8765. 10.3390/ijms21228765 33233496 PMC7699575

